# Astrocytic spermidine insufficiency contributes to enhanced pain sensitivity associated with ApoE4

**DOI:** 10.1186/s10194-025-02054-8

**Published:** 2025-05-15

**Authors:** Kaiming Yu, Xiongyao Zhou, Baolong Li, Jialu Sun, Tuo Yang, Weizhen Li, Ningning Wang, Xiaosong Gu, Shusen Cui, Rangjuan Cao

**Affiliations:** 1 Department of Hand and Foot Surgery, China-Japan Union Hospital of Jilin University, Changchun, China; 2 Key Laboratory of Peripheral Nerve Injury and Regeneration of Jilin Province, Changchun, China; 3 Key Laboratory of Neuroregeneration of Jiangsu and Ministry of Education, Co-innovation Center of Neuroregeneration, Nantong University, Nantong, Jiangsu China

**Keywords:** ApoE4, Neuropathic pain, Astrocyte, Spermidine, NOS2

## Abstract

Neuropathic pain is a chronic condition with limited effective treatments, closely associated with astrocytes and their role in central sensitization. Apolipoprotein E (ApoE), predominantly expressed in astrocytes in central nervous system, exists in three ApoE isoforms (ApoE2, ApoE3, and ApoE4) in humans, with ApoE4 linked to increased susceptibility to neurological diseases. However, the relationship between ApoE4 and neuropathic pain, as well as underlying mechanisms, remains poorly understood. Here, we demonstrated that mice expressing human ApoE4 (ApoE4-TR) displayed increased pain sensitivity following spared nerve injury (SNI) compared to ApoE3-TR mice. This increased sensitivity was also observed in mice with astrocyte-specific expression of ApoE4, achieved through Cre-mediated recombination. Metabolomic profiling revealed reduced spermidine levels in the spinal dorsal horn of ApoE4-TR mice relative to ApoE3-TR mice. Daily gavage administration of spermidine alleviated mechanical pain to a comparable level in ApoE3-TR and ApoE4-TR mice, as assessed by von Frey test. However, lower dose of spermidine effectively alleviated neuropathic pain in ApoE3-TR mice but showed reduced efficacy in ApoE4-TR mice, likely due to limited spermidine retention in ApoE4 astrocytes, as demonstrated in vitro. Transcriptomic analysis identified *Nos2* as a critical gene upregulated in ApoE4-TR mice. Mechanistically, spermidine suppressed *Nos2* expression by inhibiting the NF-κB pathway in astrocytes, thereby alleviating neuropathic pain. These findings highlight an enhanced pain sensitivity associated with ApoE4 and suggest spermidine as a potential therapeutic strategy, emphasizing a tailored dosage approach for ApoE4 carriers.

**Supplementary Information:**

The online version contains supplementary material available at 10.1186/s10194-025-02054-8.

## Introduction

Peripheral nerve injury-induced neuropathic pain is a prevalent, persistent, and debilitating health issue [1]. Spinal cord glial cells, including astrocytes and microglia, play a crucial role in the onset and progression of neuropathic pain [2, 3]. Astrocytes, the main glial cells in the central nervous system (CNS), significantly influence the induction and maintenance of central sensitization through the secretion of inflammatory cytokines and chemokines [4, 5]. Through their interactions with neurons and other glial cells, astrocytes drive neuroinflammatory responses that enhance neuronal excitability, thereby amplifying pain sensitivity in conditions like neuropathic pain [6, 7]. Therefore, identifying the molecular mechanisms underlying astrocyte involvement in central sensitization may help develop novel treatment strategies for neuropathic pain.

Apolipoprotein E (ApoE), a key lipid homeostasis regulator, is primarily expressed by astrocytes within the spinal cord and brain. ApoE has three allelic variants in humans, which differ at two amino acid positions (112 and 158): ApoE2 (Cys112, Cys158), ApoE3 (Cys112, Arg158), and ApoE4 (Arg112, Arg158) [8]. Whereas, mice have only one ApoE allele [9]. Research has established connections between ApoE and neuroinflammation, as well as synaptic plasticity, within the nervous system [10]. ApoE isoforms correlate with varied susceptibilities to neurological diseases [11–13]. Specifically, ApoE4, present in approximately 13.3% of the population, is identified as the strongest genetic risk factor for late-onset Alzheimer’s Disease (AD) [14] and is associated with increased severity of neuropathy in diabetic patients [15, 16]. Recently, ApoE has been linked to chronic pain through single-cell RNA sequencing studies [17] and to inflammatory pain regulation [18]. However, whether ApoE4 mice exhibit different pain sensitivity compared to other ApoE isoforms after nerve injury, as well as underlying mechanisms, remains unclear.

In this study, using ApoE-targeted replacement (TR) mice, which express human ApoE3 or ApoE4 under the control of the endogenous murine ApoE promoter, we demonstrated that ApoE4-TR mice exhibited increased mechanical pain sensitivity compared to ApoE3-TR mice following spared nerve injury (SNI). Since ApoE is predominantly expressed in astrocytes within the CNS, AAV-GFAP-EGFP-Cre was delivered to the spinal dorsal horn of LSL-hApoE3 or LSL-hApoE4 mice to specifically induce hApoE3 or hApoE4 expression in astrocytes. Consistently, increased pain sensitivity was observed in LSL-hApoE4 mice, suggesting that the enhanced pain sensitivity is modulated, at least in part, by ApoE4-expressing astrocytes. Metabolomic profiling of spinal dorsal horn revealed reduced spermidine levels in ApoE4-TR mice related to ApoE3-TR mice. Spermidine is a kind of polyamine primarily stored in astrocytes within the CNS, where it plays a role in protecting against oxidative stress. Therefore, spermidine was chosen for further investigation. Gavage administration of spermidine attenuated neuropathic pain in both genotypes. However, a half-dose of spermidine effectively alleviated mechanical pain in ApoE3-TR mice but exhibited diminished efficacy in ApoE4-TR mice. Further studies indicated limited spermidine retention in ApoE4-expressing astrocytes, which thereby required a higher dose for therapeutic effect. Transcriptomic analysis identified *Nos2* as a key gene differentially expressed between ApoE3-TR and ApoE4-TR mice. Additionally, spermidine was found to modulate *Nos2* expression via the NF-κB pathway to achieve analgesic effects. These findings not only elucidate the enhanced pain sensitivity associated with ApoE4 isoform but also highlight spermidine as a promising therapeutic candidate for neuropathic pain management. Specifically, individuals with the ApoE4 genotype may require higher doses of spermidine to achieve comparable pain relief.

## Methods

### Ethics statement

All animal experiments were conducted in accordance with the US National Institute of Health (NIH) Guide for the Care and Use of Laboratory Animals, following the guidelines of the International Association for the Study of Pain (IASP) for animal research, and was approved by the Jilin University Administration Committee of Experimental Animals (Approval No. 20230611-01). The mice were maintained on a standard diet with ad libitum access to water under controlled room temperature (~ 25 °C) and a 12-h light/dark cycle. Pain induction levels were kept within ethical and scientific standards, and all necessary measures were taken to balance the scientific objectives with animal welfare. Animals displaying severe distress, such as a ≥ 20–25% weight loss, lethargy, loss of appetite, difficulty breathing, self-mutilation, infection, or other severe health conditions, were humanely euthanized following the AVMA Guidelines for the Euthanasia of Animals (using cervical dislocation). All mice were randomly assigned to experimental groups and sacrificed no later than 28 days post-surgery.

### Mice

Human ApoE3-targeted replacement (ApoE3-TR) (B6.Cg-ApoE^em2(ApoE*)Adiuj^/J, #029018) and ApoE4-TR (B6(SJL)-ApoE^tm1.1(ApoE*4)Adiuj^/J, #027894) mice were from the Jackson Laboratory. The R26-CAG-LSL-hApoE3-3xFlag (LSL-hApoE3) and R26-CAG-LSL-hApoE4-3xFlag (LSL-hApoE4) mice were generated utilizing CRISPR/Cas9 technology to insert the CAG-LSL-hApoE3-3xFlag-Wpre-pA or CAG-LSL-hApoE4-3xFlag-Wpre-pA expression cassette into the Rosa26 gene locus via homologous recombination by Shanghai Model Organisms Center, Inc. Briefly, Cas9 mRNA and gRNA were synthesized through in vitro transcription. The donor vector was constructed by in-fusion cloning, with the plasmid containing a 5’ homologous arm (3.3 kb), the knock-in sequence, and a 3’ homologous arm (3.3 kb). A mixture of Cas9 mRNA, gRNA, and the donor vector was then microinjected into fertilized C57BL/6J (Shanghai Model Organisms Center, Inc) eggs. Three F0 mice were identified through long-range PCR, and a positive F0 mouse was crossed with wild-type C57BL/6J mice to generate F1 offspring. Sanger sequencing was performed on PCR products from F1 mice to confirm accurate on-target insertion without indels or mutations at the junctions.

### Quality control of transgenic mice

For both ApoE3-TR and ApoE4-TR mice, breeding pairs were validated by Sanger sequencing to confirm the presence of ApoE3-specific (T/C) or ApoE4-specific (C/C) single nucleotide polymorphisms at amino acid positions 112 and 158, respectively. To ensure correct allele inheritance and genetic consistency, all experimental offspring were genotyped, and only homozygous mice were used in subsequent experiments (Fig. S1A-D). For LSL-ApoE3 and LSL-ApoE4 mice, genotyping was performed to identify homozygous individuals, which were selected for subsequent experiments (Fig. S1E).

### Spared nerve injury

Male mice at the ages of 8–10 weeks (22–26 g) were deeply anesthetized with 2% isoflurane and subjected to unilateral spared nerve injury (SNI) or sham surgeries as described previously [19]. For the SNI procedure, the left sciatic nerve was exposed by making an incision on the skin of the lateral surface of the mouse thigh and sectioning through the biceps femoris muscle. Two of the three terminal branches of the sciatic nerve, the tibial and common peroneal nerves, were tightly ligated with 6 − 0 silk (Covidien, S1768K), and 2–4 mm of the distal nerve segments were removed while ensuring the sural nerve remained undisturbed. The muscle and skin were then closed using coated Vicryl sutures (Ethicon, J489G). In sham surgeries, the sciatic nerve and its three branches were exposed but left intact. Carprofen (HY-B1227, MedChemExpress) at the concentration of 5 mg/kg was administered once daily for 2 consecutive days after surgery via subcutaneous injection to all mice undergoing surgical procedures.

A total of 75 male ApoE3-TR mice and 75 male ApoE4-TR mice, as well as 25 male LSL-hApoE3 mice and 25 male LSL-hApoE4 mice, were used in the study. Each genotype was equally divided between the SNI and sham groups.

### Mechanical pain test using von frey filaments

All experiments were conducted during the light cycle, between 09:00 am and 16:00 pm. Mice that underwent unilateral SNI were placed in custom-made Plexiglas cubicles (5.3 × 8.5 × 3.6 cm) with a perforated metal floor and habituated for at least 1 h before testing. Mechanical paw withdrawal threshold (PWT) sensitivity was assessed using calibrated von Frey filaments. The filaments were applied perpendicularly to the plantar surface of the hind paw with sufficient force to induce bending. A withdrawal response occurring within 4 s was recorded as a positive response. If the initial filament did not elicit a response, the next filament with a higher force was tested. Conversely, if a response was observed, a filament with a lower force was applied instead. Von Frey filaments ranging from 0.04 g to 1.4 g were used in this study. At least two consecutive measurements were taken on each hind paw at each time point and averaged. Mice were tested for mechanical sensitivity at baseline and 7, 14, and 28 days after surgery to quantify mechanical allodynia.

### DigiGait™ analysis

DigiGait™ is equipped with a transparent treadmill, where animals are placed under a polymethyl methacrylate cover and forced to walk or run at a fixed velocity (10 cm/s) and gradient (0°). Before the experiments, animals were trained to complete uninterrupted runs for at least 3 step cycles at a speed of at least 10 cm/s. Measurements were taken before SNI or 14 days after SNI surgery. Each mouse was placed on the treadmill at intervals of at least 5 min to complete three uninterrupted runs (each containing at least 3 step cycles) for further analysis. Consecutive recordings (150 fps) from a ventral direction provided the following coordination parameters: DigiGait-swing and DigiGait-duty cycle. DigiGait-swing refers to the duration of the swing phase during walking, while DigiGait-duty cycle is the ratio of time spent in the stance phase to the time spent in a single step.

### Primary mouse astrocyte isolation and culture

Mixed glial cultures, comprising microglia and astrocytes, were isolated from the spinal cords of postnatal 0 to 1-day-old ApoE3-TR or ApoE4-TR mouse pups, as previously described [20]. Briefly, whole spinal cords were collected, washed in 0.01 M PBS, treated with 0.1% trypsin, thoroughly triturated, and filtered with a 70 μm strainer. The collected cells were grown in DMEM, supplemented with 2 mM Glutamax, 1 mM sodium pyruvate, 20 mM HEPES, and 10% FBS (S-FBS-SA-015, Serana). Purified astrocytes were obtained by overnight shaking to remove oligodendrocyte progenitor cell, followed by sequential panning on non-tissue culture-treated plastic to eliminate microglia. The cells were then passaged in DMEM (C11995500CP, Gibco), supplemented with 10% FBS and Penicillin-Streptomycin (15140-122, Gibco) on PLL-coated plates, and cultured at 37 °C in a humidified incubator with 5% CO2.

### Cell treatments

In the experiments, primary astrocytes were seeded at a density of 30,000 cells per well in 96-well plates and at a density of 70,000 cells per well in 24-well plates. For each isolation, cells were seeded in two different plates, with three replicates for each treatment group per plate. Primary cells were treated with 5 ng/ml IL-1β (211-11B, ThermoFisher), 10 ng/ml TNF-α (315–01 A, ThermoFisher), and 10 ng/ml IFN-γ (315-05, ThermoFisher). The NF-κB activator phorbol-12-myristate-13-acetate (PMA) (HY-18739, MedChemExpress) was dissolved in DMSO. Primary astrocytes were incubated with medium containing 100 nM PMA for the indicated periods, with medium containing DMSO as a control.

### Conditioned media collection

Primary astrocytes were treated with cytokine mix (CYM) (IL-1β at 5 ng/ml, TNF-α at 10 ng/ml, and IFN-γ at 10 ng/ml) for 6 h. After treatment, the cells were washed three times with fresh media and replaced with new media. Cytokines were allowed to accumulate for the indicated periods, after which the media was collected and stored in aliquots at -80 °C for further ELISA analysis.

### Immunostaining

Approximately 30,000 astrocytes were seeded per well in 12-well plates with coverslips coated by poly-L-lysine (PLL). Once the cells reached ~ 80% confluence, they were washed with 0.01 M PBS and fixed with 4% paraformaldehyde (PFA) for 10 min. The fixed cells were washed twice with PBS, followed by blocking with a solution containing 2% bovine serum albumin (BSA) and 0.3% Triton X-100 in PBS for 30 min.

For tissue samples, mice were deeply anesthetized with isoflurane and transcardially perfused with PBS, followed by 4% PFA until limb stiffness was observed. The L4/5 lumbar spinal cord was then carefully isolated and fixed with 4% PFA at 4 °C overnight. After fixation, the tissue was dehydrated in 30% sucrose at 4 °C until it sank to the bottom. Subsequently, the tissue was embedded in OCT compound and sectioned at a thickness of 30 μm using a cryostat (Leica CM1950, Germany). The sections were then air-dried and washed three times with PBS. Blocking was carried out using 5% BSA and 1% Triton X-100 in PBS for 30 min. Primary antibodies diluted in blocking buffer (1:500) were applied and incubated overnight at 4 °C at the following concentrations: ApoE (Cell Signaling Technology, Cat# 13366, RRID: AB_2798191), GFAP (Millipore, Cat# MAB360, RRID: AB_11212597), NeuN (Cell Signaling Technology, Cat# 24307, RRID: AB_2651140), and Iba1 (Abcam, Cat# ab5076, RRID: AB_2224402). On the following day, samples were washed with PBS and incubated with appropriate secondary antibodies (1:1000) for 1 h at room temperature. The following secondary antibodies are used: Goat anti-Mouse IgG conjugated with Alexa Fluor™ 488 (Thermo Fisher Scientific, Cat# A-11001, RRID: AB_2534069), Donkey anti-Rabbit IgG conjugated with Alexa Fluor™ 647 (Thermo Fisher Scientific, Cat# A-31573, RRID: AB_2536183), Donkey anti-Goat IgG conjugated with Alexa Fluor™ 647 (Thermo Fisher Scientific, Cat# A-21447, RRID: AB_2535864), Goat anti-Rabbit IgG conjugated with Alexa Fluor™ 546 (Thermo Fisher Scientific, Cat# A-11035, RRID: AB_2534093) and Goat anti-Mouse IgG conjugated with Alexa Fluor™ 546 (Thermo Fisher Scientific, Cat# A-11030, RRID: AB_2737024).

If necessary, nuclei were counterstained with DAPI (1:1000, Thermo Fisher Scientific, Cat# D3571, RRID: AB_2307445). Images were acquired using a confocal microscope (Nikon A1, Japan) equipped with either a 20× or 10× objective. Lasers at 405 nm, 488 nm, 546 nm, and 647 nm were used to excite DAPI, Alexa Fluor 488 or EGFP, Alexa Fluor 546, and Alexa Fluor 647, respectively. The images were analyzed using ImageJ software to calculate the mean fluorescence intensity for each respective channel.

### Western blot

Tissue and cell samples were lysed with RIPA buffer (C500008, Sangon Biotech) containing a mixture of protease inhibitors and phosphatase inhibitors. The lysis was then purified by centrifugation at 4 °C for 30 min at 12,000 rpm. Protein concentrations were determined using the bicinchoninic acid assay (P0010, Beyotime Biotechnology). Protein samples were separated by 10% SDS-PAGE and transferred onto PVDF membranes. The membrane was blocked with 5% nonfat milk for 1 h at room temperature and then incubated with primary antibody at 4 °C overnight. After washing with 0.1% Tween in TBS three times (10 min/each), the membrane was incubated with HRP-conjugated secondary antibodies for 1 h at room temperature. The following antibodies were used: Flag (1:2000, Cell Signaling Technology, Cat# 14793, RRID: AB_2572291), NF-κB p65 (1:1000, Cell Signaling Technology, Cat# 8242, RRID: AB_10859369), Phospho-NF-κB (1:1000, Cell Signaling Technology, Cat# 3033, RRID: AB_331284), NOS2 (1:1000, Santa Cruz Biotechnology, Cat# sc-7271, RRID: AB_627810), and β-actin (1:3000, Transgen Biotech, Cat# HC201, RRID: AB_2860007). ApoE, GFAP, and Iba1 antibodies were used at a dilution of 1:1000, and their information is detailed above. The images were scanned using a GS800 Densitometer Scanner, and blotting intensities were quantified using Fuji software (Tokyo, Japan). Western blot was repeated three times for the statistical analysis. The intensities of the target proteins were normalized to those of β-actin.

### Spinal injection of virus

Briefly, mice were deeply anesthetized with 2% isoflurane, and the paraspinal muscles around the left T13-L1 intervertebral space were carefully removed. A 30G needle was used to create a small incision in the dura mater and arachnoid membrane, forming a window for the insertion of a glass micro-pipette (80 μm in diameter) into the superficial dorsal horn (SDH). The mice were secured in a spinal stabilizer, and an AAV viral solution was injected through the window, approximately 500 μm lateral to the midline.The micro-pipette was inserted into the SDH at a depth of 250 μm from the surface of the dorsal root entry zone, and 0.6 µl of the solution was delivered in a single injection for each mouse. AAV-hSyn-GCaMP6f (5.01 × 10^12^ vg/ml, BC-0079), AAV-GFAP-EGFP-Cre (1.23 × 10^13^ vg/ml, BC-1363), and AAV-GFAP-Cre (1.12 × 10^13^ vg/ml, BC-0166) were all obtained from Brain Case, China.

### Fiber photometry-based calcium imaging

AAV-hSyn-GCaMP6f virus was injected into the superficial layers of the spinal dorsal horn to drive GCaMP6f expression in dorsal horn neurons. Fourteen days after the SNI model, mice were fixed in a spinal stabilizer, the L3 vertebra was exposed, and the vertebral column on the modeling side was drilled open using a cranial drill. An optical fiber (250 μm O.D., 0.37 NA) was placed into a ceramic ferrule and secured to a stereotaxic frame. The optical fiber was then implanted into the superficial layer (1/2) of the spinal cord (250 μm in depth from the surface of the dorsal root entry zone). Mice with inaccurate injection sites were excluded from further experiments. Calcium transients were captured using a fiber photometry system (R821, RWD Life Science Co., Ltd). GCaMP6f fluorescence was excited with a 470 nm LED (30 µW at the fiber tip), while calcium-independent signals were excited with a 410 nm LED (20 µW at the fiber tip), with both LEDs alternating at 20 Hz. Emission signals were recorded using an sCMOS camera (Photo-metrics Prime) at matching frequency [21]. Pain was induced by stimulating the lateral plantar region of the injury side with 0.4 g von Frey nylon monofilaments, and calcium signals in the spinal dorsal horn were recorded. Signal quantification involved subtracting the calibrated 410 nm signal from the 470 nm signal to correct for movement and bleaching. The ΔF/F ratio was calculated as (470 nm signal - calibrated 410 nm signal) / (calibrated 410 nm signal) -1, and further normalized using the pre-exposure window from − 1 to 0 s as the baseline period. The area under the curve (AUC) in response to exposure from 0 to 5 s was then calculated.

### Real-time quantitative polymerase chain reaction (RT-qPCR)

Total RNA was extracted using the Eastep™ Super Total RNA Extraction Kit (LS1040, Promega), and cDNA synthesis was performed using the TransScript One-Step cDNA Synthesis SuperMix (AT311, TransGen Biotech). Primers were synthesized by Genewiz Biotech. cDNA templates and primers were mixed with TB Green™ Premix Ex Taq™ (RR420A, TaKaRa), and real-time quantitative PCR was conducted using the Real-Time PCR System (CFX96, Bio-Rad). Relative gene expression was calculated using the 2^−ΔΔCt^ method, with β-actin serving as an endogenous control. The sequences of the primers used in this study are as follows: IL-1β, forward: TGGAC CTTCC AGGAT GAGGA CA, reverse: GTTCA TCTCG GAGCC TGTAG TG; IL-6, forward: TACCA CTTCA CAAGT CGGAG GC, reverse: CTGCA AGTGC ATCAT CGTTG TTC; TNF-α, forward: GGTGC CTATG TCTCA GCCTC TT, reverse: GCCAT AGAAC TGATG AGAGG GAG; Nos2, forward: GAGAC AGGGA AGTCT GAAGC AC, reverse: CCAGC AGTAG TTGCT CCTCT TC; Sgk1, forward: CTCAT TCCAG CCGCT GACAA AC, reverse: CCAAG GCACT GGCTA TTTCA GC; Mfap4, forward: TCAGG AAGAC GGCGT GTATC TC, reverse: GAAAC TCACT GAGCC GTTGA ATC; Vegfd, forward: CTCCA CCAGA TTTGC GGCAA CT, reverse: ACTGG CGACT TCTAC GCATG TC; Pcsk9, forward: ATGGC ACCAG ACAGA GGAAG AC, reverse: CACGC TGTTG AAGTC GGTGA TG; S100a8, forward: CAAGG AAATC ACCAT GCCCT CTA, reverse: ACCAT CGCAA GGAAC TCCTC GA; β-actin, forward: CATTG CTGAC AGGAT GCAGA AGG, reverse: TGCTG GAAGG TGGAC AGTGA GG.

### Enzyme-linked immunosorbent assay (ELISA) for cytokine detection

The assessment of IL-1β (E-EL-M0037), IL-6 (E-EL-M0044), and TNF-α (E-EL-M3063) was performed using specific mouse ELISA kits from Elabscience Biotechnology of China. The assessment of spermidine (LV30616) was conducted using kits from Animalunion Biotechnology of China. Briefly, 100 µl of standards, samples, and controls were added to the wells and incubated at 37 °C for 90 min. After washing, 100 µl of biotinylated detection antibody was added and incubated for 1 h, followed by 100 µl of HRP-conjugate for 30 min. After five additional washes, 90 µl of substrate solution was added and incubated for 15 min in the dark. The reaction was terminated with 50 µl of stop solution, and absorbance was measured at 450 nm. Cytokine and spermidine concentrations were determined from standard curves.

### Metabolomics analysis using UHPLC-MS/MS

UHPLC-MS/MS analyses were performed using a Vanquish UHPLC system (Thermo Fisher, Germany) coupled with an Orbitrap Q Exactive™ HF mass spectrometer (Thermo Fisher, Germany) in Novogene Co., Ltd. (Beijing, China). Samples were injected onto a Hypesil Goldcolumn (100 × 2.1 mm, 1.9 μm) using a 17-min linear gradient at a flow rate of 0.2 ml/min. The raw data files generated by UHPLC-MS/MS were processed using the Compound Discoverer 3.3 (CD3.3, ThermoFisher) to perform peak alignment, peak picking, and quantitation for each metabolite. Statistical analyses were performed using the statistical software R (R version R-3.4.3), Python (Python 2.7.6 version) and CentOS (CentOS release 6.6), When data were not normally distributed, standardize according to the formula: sample raw quantitation value / (The sum of sample metabolite quantitation value / The sum of QC1 sample metabolite quantitation value) to obtain relative peak areas. And compounds whose CVs of relative peak areas in QC samples were greater than 30% were removed, and finally the metabolites’ identification and relative quantification results were obtained. These metabolites were annotated using the KEGG database, HMDB database, and LIPIDMaps database. Principal Component Analysis (PCA) and Partial Least Squares Discriminant Analysis (PLS-DA) were performed using metaX, a flexible and comprehensive metabolomics data processing software. Univariate analysis (t-test) was applied to calculate statistical significance (*P* values). Metabolites with VIP > 1, *P* < 0.05, and fold change ≥ 2 or FC ≤ 0.5 were considered differentially expressed metabolites.

### Measurement of spermidine content

The content of spermidine was measured by high-performance liquid chromatography (HPLC) using an UltiMate 3000 HPLC system (Thermo Fisher Scientific), following the benzoylation procedure described previously. A 20 µl sample of benzoylated polyamines was separated on a 5 μm particle size C18 column (4.6 × 250 mm, Agilent Technologies). The mobile phase consisted of methanol (A) and water (B) in a ratio of 62:38. Isocratic elution was performed as follows: 17 min, 62% mobile phase A. The column temperature was maintained at 25 °C, and the flow rate was 1 ml/min. Polyamine peaks were detected using a fluorescence detector at 229 nm. Results were compared to the standard curve (Sigma-Aldrich).

### Spermidine treatment

For the in vivo experiments, sterile-filtered spermidine solution prepared in PBS (44.7 mM or 22.35 mM) was administered daily via oral gavage (200 µl) starting from the first day after SNI surgery. For the in vitro experiments, treatment was conducted by adding spermidine dissolved in PBS at final concentrations of 10 mM or 5 mM.

### RNA sequencing

The spinal cord of mice was quickly removed, accurately weighed, placed in 1.5 ml EP tubes, and frozen at -80 °C until analysis. RNA was extracted from the spinal cord tissues of mice using TRIzol™ reagent (15596-026, ThermoFisher). The total amount and integrity of the spinal cord RNA were assessed using the Agilent Technologies Bioanalyzer 2100 system. The construction of the cDNA library, quality inspection, clustering, and sequencing were conducted according to the procedures of Novogene (Beijing, China). A *P*-value < 0.05 was considered as the criterion for evaluating differentially expressed genes (DEG). In addition, gene set enrichment analysis (GSEA) was performed on the GSEA 4.1.0 software with gene sets sourced from the MSigDB database. The pathway and function annotation of DEG was performed through co-enrichment analysis using the Kyoto Encyclopedia of Genes and Genomes (KEGG) and Gene Ontology (GO).

### Total NO production assay

Total NO production was estimated by measuring the accumulation of nitrite and nitrate in the spinal cord dorsal horn or cells using the Griess reagent via the Total Nitric Oxide Assay Kit (S0023, Beyotime). Nitrate was measured after enzymatic conversion to nitrite by nitrate reductase. Nitrite is the end stable product of NO metabolism. Briefly, 60 µl of each sample supernatant was mixed with an equal volume of dilution buffer and added to duplicate wells of a 96-well plate at room temperature. The mixture was incubated at 37 °C for 15 min with 5 µl of nicotinamide adenine dinucleotide phosphate (NADPH), 10 µl of flavin adenine dinucleotide (FAD), and 5 µl of nitrate reductase. Then, 10 µl of lactate dehydrogenase (LDH) buffer and 10 µl of LDH were added to the reaction buffer and incubated for another 5 min at 37 °C. Finally, 50 µl of Griess reagent I and 50 µl of Griess reagent II were added to all wells and incubated for 10 min. Optical density at 540 nm was measured using an OPTImax multiplate reader. Concentrations were calculated by comparing the absorbance values to a standard curve (50, 20, 10, 5, and 2 µM sodium nitrite).

### Intrathecal injection of PMA

PMA was diluted to a concentration of 5 µg/ml. The mice were anesthetized with 2% isoflurane in a prone position on the operating table. The bilateral iliac bones were fixed, and the L5-L6 intervertebral space was identified as the injection site. A sudden tail flick was used as an indicator of successful intervertebral insertion. Then, 20 µl of the PMA diluent was injected using a U40 insulin needle (kdl, 0.3 × 8 mm) at a rate of 1 µl per 4 s. After the injection, the needle position was maintained for approximately 3 min, followed by gentle rotation and withdrawal of the syringe to avoid leakage.

### Statistical analysis

All data in this study were expressed as mean ± SEM, and analyzed using GraphPad Prism Software 8.4.2 (La Jolla, CA). For comparisons between two groups, unpaired Student’s t-tests were applied. For multiple group comparisons, one-way ANOVA was performed for single-factor comparisons, with Tukey post hoc analyses applied where appropriate. Two-way ANOVA was used when two independent variables were involved, followed by Bonferroni post hoc analyses. Each experiment included at least three biological replicates, and data were assumed to follow a normal distribution without testing. For the behavior test, Cohen’s d was calculated and effect size interpreted as small (0.2–0.5), medium (0.5–0.8), or large (> 0.8) [22]. *P < 0.05 was considered statistically significant, with additional levels of significance indicated as **P < 0.01, ***P < 0.001, ****P < 0.0001.

## Results

### ApoE4-TR mice exhibited increased sensitivity to neuropathic pain

**Fig. 1:**
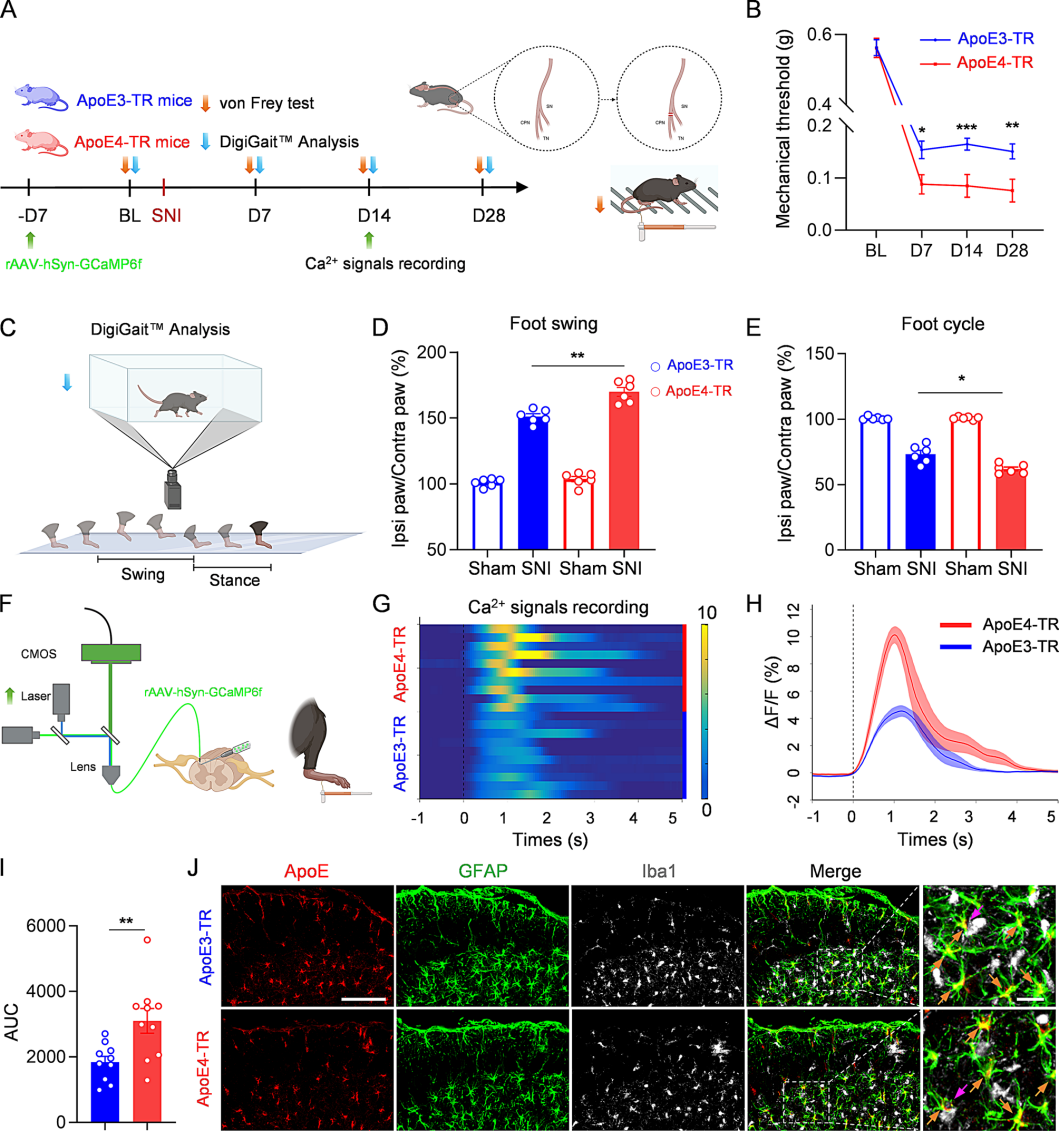
ApoE4 enhanced neuropathic pain sensitivity in mice. **(A)** Schematic of experimental design, SNI surgery, and von Frey filament test. **(B)** Mechanical thresholds of ApoE3-TR mice and ApoE4-TR mice before and after SNI. *n* = 10 mice per group. Cohen’s d for ApoE3-TR vs. ApoE4-TR post-SNI: 1.26 (D7), 1.46 (D14), 1.29 (D28). **(C)** Schematic diagram of DigiGait™ analysis. **(D**,** E)** Quantification of foot swing **(D)** and foot cycle **(E)** during DigiGait™ analysis of ApoE3-TR mice and ApoE4-TR mice before and 14 days after SNI. *n* = 6 mice per group. Cohen’s d = 2.71 for foot swing and 2.06 for foot cycle (ApoE3-TR SNI vs. ApoE4-TR SNI). **(F)** Schematic diagram of mechanical pain-evoked Ca^2+^ signals recording. CMOS, complementary metal oxide semiconductor. **(G)** Heatmap showing the GCaMP6f fluorescence changes in the superficial dorsal horn of the spinal cord in ApoE3-TR and ApoE4-TR mice following pain stimulation 14 days after SNI. *n* = 10 mice per group. **(H)** Peri-event plot of GCaMP6f ΔF/F after pain stimulation. **(I)** Quantification of the mean AUC value for ApoE3-TR and ApoE4-TR mice. *n* = 10 mice per group. **(J)** Representative images of the spinal dorsal horn labeled with anti-ApoE (red), anti-GFAP (green), and anti-Iba1 (gray) antibodies in ApoE3-TR and ApoE4-TR mice 14 days after SNI. Scale bar, 30 μm (left), 10 μm (right). All data are expressed as mean ± SEM. Statistical comparisons were conducted with two-way ANOVA followed by Bonferroni’s post hoc test **(B)**; one-way ANOVA followed by Tukey’s post hoc test **(D, E)**; and unpaired Student’s t-test **(I)**. *P < 0.05, **P < 0.01, and ***P < 0.001

Since ApoE4 is linked to multiple neurological diseases, we examined whether ApoE4 mice exhibit different pain sensitivity compared to other ApoE isoforms after nerve injury. Given that ApoE3 is present in approximately 79% of the human population [14, 23], we compared the mechanical pain thresholds between ApoE3-targeted replacement (ApoE3-TR) mice and ApoE4-TR mice by von Frey filament test after spared nerve injury (SNI) (Fig. 1A). Intriguingly, the mechanical pain threshold of ApoE4-TR mice was significantly lower than that of ApoE3-TR mice from as early as 7 days post-injury, and even much lower at days 14 and 28 (Fig. 1B). Mice were also subjected to DigiGait™ analysis (Fig. 1C), which objectively captures pain- and motor dysfunction-induced changes in various walking parameters and has been successfully applied to evaluate non-reflexive pain behaviors [17]. Compared to ApoE3-TR mice, ApoE4-TR mice showed a higher swing phase and a lower duty cycle, highlighting their enhanced pain sensitivity in non-reflexive pain behaviors (Fig. 1D, E).

Next, we compared the neuron activity between the two groups post-SNI by fiber photometry recording. One week before the surgery, AAV-hSyn-GCaMP6f was injected into the superficial dorsal horn (layer I/II) of the spinal cord of ApoE3-TR or ApoE4-TR mice (Fig. 1F). On day 14 post-surgery, the mice were immobilized and changes in Ca^2+^ signals (measured via fluorescence) were recorded when stimulating the left hind paw. Consistent with the behavioral findings, Ca^2+^ transients in neurons of superficial dorsal horn were elevated in ApoE4-TR mice (Fig. 1G, H). The area under the curve (AUC) for Ca^2+^ signaling was significantly larger in ApoE4-TR mice compared to ApoE3-TR mice (Fig. 1I), indicating stronger neuronal activity in ApoE4-TR mice. Following that, the accuracy of the virus injection site was confirmed through immunohistochemistry staining (Fig. S2A, B), and viral transduction efficiency and expression levels were comparable between ApoE3-TR and ApoE4-TR mice (Fig. S2C, D).

Since the activation of astrocytes or microglia plays a crucial role in the onset and progression of neuropathic pain [1, 3, 4], we investigated whether the observed differences in hypersensitivity were associated with differential glial activation between the two genotypes by immunostaining. Results showed that both ApoE3 and ApoE4 mainly co-localized with GFAP-positive astrocytes, with minimal expression in microglia (Fig. 1J). At 14 days post-SNI, astrocytes and microglia were activated in both genotypes compared with their sham groups (Fig. S2E), aligning with earlier studies [24, 25]. However, no significant differences were observed in the fluorescence intensity of ApoE, GFAP or Iba1 staining (Fig. S2F). Western blot analysis showed that the expression levels of GFAP, Iba1, and ApoE were significantly upregulated in both ApoE3-TR and ApoE4-TR mice following SNI compared to their respective sham controls. However, these proteins were comparable between ApoE3-TR and ApoE4-TR mice under both sham and SNI conditions (Fig. S2G-J). These findings suggested that ApoE4-TR mice were more sensitive to neuropathic pain, probably due to the increased hypersensitivity of neurons in layer I/II after nerve injury.

### Astrocytic ApoE4 in spinal cord contributed to the enhanced pain sensitivity

**Fig. 2:**
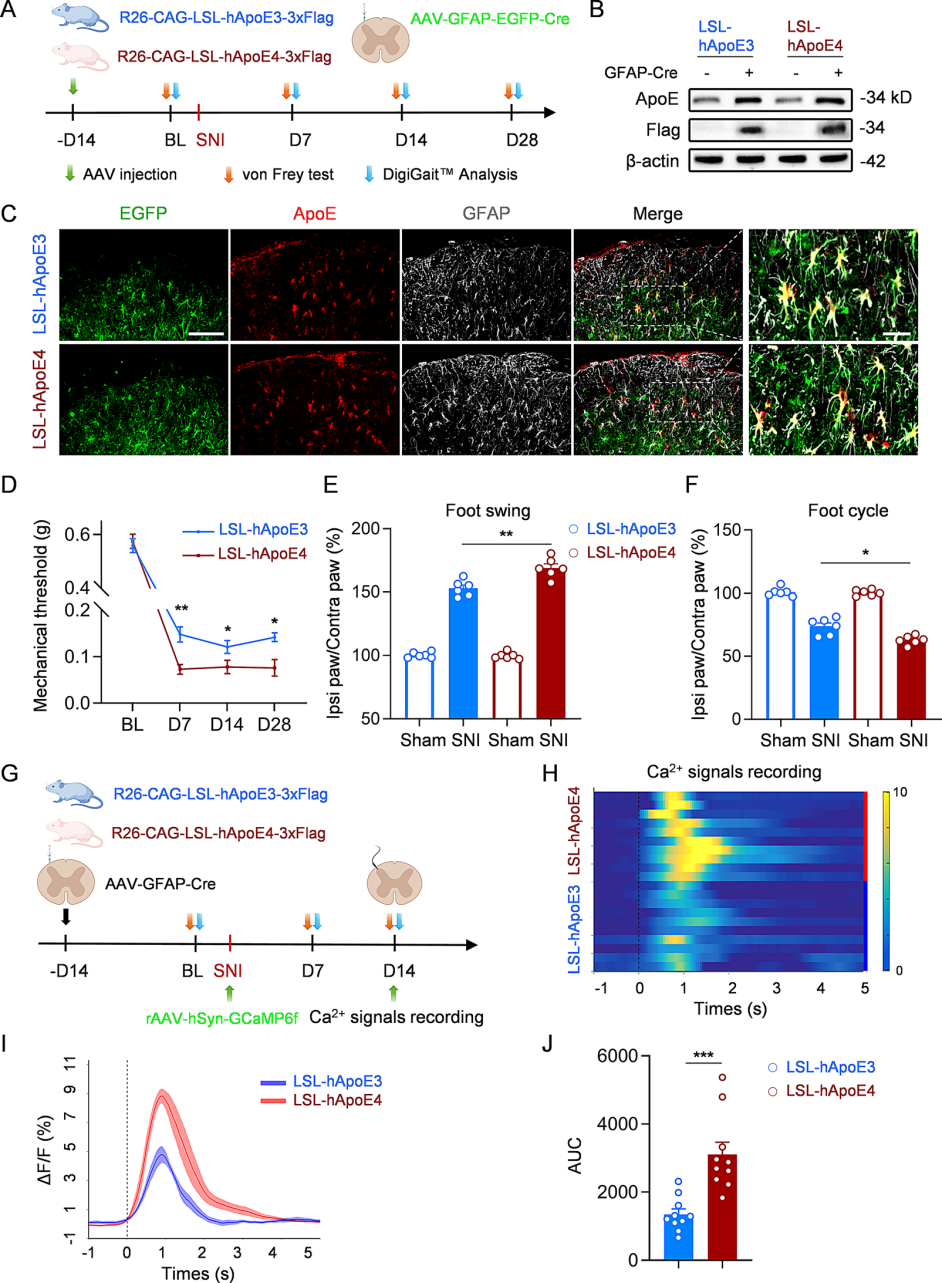
Astrocytic ApoE4 in spinal dorsal horn contributed to the increased pain sensitivity. **(A)** Schematic of experimental design, AAV administration, and transgenic mice information. **(B)** Western blots showing the expression of Flag and ApoE after the injection of AAV-GFAP-EGFP-Cre virus. *n* = 3 independent biological replicates per group. **(C)** Representative images of the spinal dorsal horn labeled with anti-ApoE (red) and anti-GFAP (gray) in LSL-hApoE3 and LSL-hApoE4 mice injected with AAV-GFAP-EGFP-Cre virus. Scale bar, 50 μm (left), 10 μm (right). **(D)** Mechanical thresholds before and after SNI in LSL-hApoE3 and LSL-hApoE4 mice following virus injection. *n* = 6 mice per group. Cohen’s d for LSL-hApoE3 vs. LSL-hApoE4 post-SNI: 1.74 (D7), 0.96 (D14), 1.48 (D28). **(E**,** F)** Quantification of foot swing **(E)** and foot cycle **(F)** during DigiGait™ analysis of LSL-hApoE3 mice and LSL-hApoE4 mice after viral injection, before and 14 days after the SNI. *n* = 6 mice per group. Cohen’s d = 2.26 for foot swing and 2.28 for foot cycle (LSL-hApoE4 SNI vs. LSL-hApoE4 SNI). **(G)** Experimental scheme of the fiber photometry analysis. **(H)** Heatmap of mechanical pain-evoked Ca^2+^ signals from LSL-hApoE3 and LSL-hApoE4 mice following injection of GFAP-Cre and GCaMP6f viruses. *n* = 10 mice per group. **(I)** Peri-event plot of GCaMP6f ΔF/F after pain stimulation. **(J)** Quantification of mean AUC value for LSL-hApoE3 and LSL-hApoE4 mice. *n* = 10 mice per group. All data are expressed as mean ± SEM. Statistical comparisons were conducted with two-way ANOVA followed by Bonferroni’s post hoc test **(D)**; one-way ANOVA followed by Tukey’s post hoc test **(E, F)**; and unpaired Student’s t-test **(J)**. *P < 0.05, **P < 0.01, and ***P < 0.001

There are studies reporting ApoE was increased in microglia under pathological conditions [17]. Consistently, our immunostaining results demonstrated increased ApoE3 and ApoE4 expression in both astrocytes and microglia. However, the upregulation was predominantly observed in astrocytes, as indicated by a markedly increased proportion of ApoE-localized astrocytes (Fig. S2K). In contrast, the percentage of ApoE-expressing microglia remained unchanged (Fig. S2L). Therefore, we focused on the roles of astrocytic ApoE3 and ApoE4 in neuropathic pain. R26-CAG-LSL-hApoE3/4-3xFlag (LSL-hApoE3 or LSL-hApoE4) mice, a genetically engineered model in which human ApoE3 (hApoE3) or hApoE4 is conditionally expressed under the control of Cre recombinase, were employed. Fourteen days before the surgery, AAV-GFAP-EGFP-Cre was injected into the superficial dorsal horn (Fig. 2A). Western blot confirmed the expression of Flag and an increase in total ApoE following viral injection (Fig. 2B). Immunostaining revealed that ApoE was primarily colocalized with GFAP and only minimally with Iba1 (Fig. 2C, S3A, B). Furthermore, over 96% of virus-infected cells were GFAP^+^ astrocytes (Fig. S3C), and no EGFP-expressing Iba1^+^ microglia were observed, suggesting that viral expression was largely restricted to astrocytes.

Next, SNI was performed in LSL-hApoE3 and LSL-hApoE4 mice receiving AAV-GFAP-EGFP-Cre virus. Von Frey test revealed no significant differences in mechanical pain thresholds between the two mice groups physiologically. However, the pain thresholds dropped consistently from day 7 up to day 28 after nerve surgery (Fig. 2D). Compared to LSL-hApoE3 mice, LSL-hApoE4 mice exhibited lower mechanical thresholds (Fig. 2D), as well as increased swing phase and reduced duty cycle in DigiGait™ analysis (Fig. 2E, F), which recapitulated earlier results observed in ApoE3/4-TR mice. To assess potential neuronal excitability differences in the dorsal horn, AAV-GFAP-Cre (without EGFP) was first injected. After 14 days, a second injection of rAAV-hSyn-GCaMP6f was administered into the superficial dorsal horn (Fig. 2G). After characterizing the pain threshold baselines, SNI was induced, and fiber photometry values were recorded in vivo at 14 days post-surgery (Fig. 2H). Similar to ApoE4-TR mice, LSL-hApoE4 mice showed higher neuronal excitability compared to LSL-hApoE3 mice (Fig. 2I, J). These findings suggested that the selective expression of hApoE4 in spinal cord astrocytes contributes to enhanced pain sensitivity.

### Elevated inflammatory cytokines in the spinal dorsal Horn of ApoE4-TR mice

**Fig. 3:**
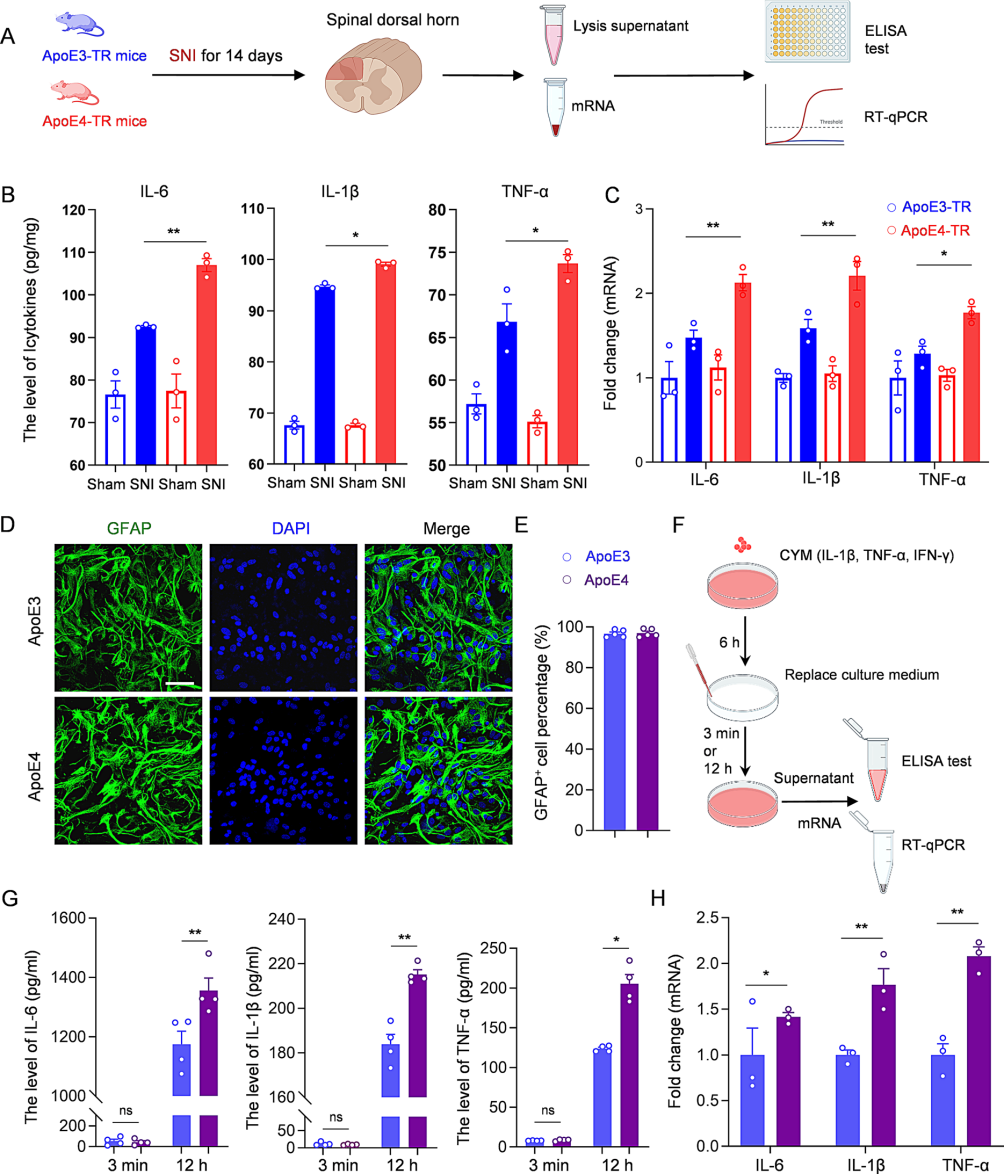
Elevated inflammatory cytokines in the spinal dorsal horn of ApoE4-TR mice. **(A)** Experimental scheme of ELISA and RT-qPCR in mice. **(B)** Quantification of cytokine levels (IL-6, IL-1β, and TNF-α) in the spinal dorsal horns of the injured side of ApoE3-TR and ApoE4-TR mice by ELISA 14 days after SNI. *n* = 3 biologically independent samples. **(C)** RT-qPCR analysis of IL-6, IL-1β, and TNF-α in the dorsal horn of ApoE3-TR and ApoE4-TR mice 14 days after SNI. *n* = 3 biologically independent samples. **(D)** Representative images of the cultured primary astrocytes stained by anti-GFAP (red) and DAPI (blue). Scale bar, 20 μm. **(E)** Quantification of GFAP^+^ cells. Each data point represents the mean percentage of GFAP^+^ cells per well, with *n* = 5 wells per group. **(F)** Experimental scheme of ELISA and RT-qPCR in cultured astrocytes. **(G)** Quantification of cytokines released from ApoE3 and ApoE4 astrocytes by ELISA at 3 min and 12 h after medium replacement. *n* = 3 biologically independent samples. **(H)** RT-qPCR analysis of IL-6, IL-1β, and TNF-α in ApoE3 and ApoE4 astrocytes after CYM treatment. *n* = 3 biologically independent samples. All data are expressed as mean ± SEM. Statistical comparisons were conducted with one-way ANOVA followed by Tukey’s post hoc test **(B, C)**; and unpaired Student’s t-test **(E, G, H)**. *P < 0.05, and **P < 0.01

Multiple studies have shown that increased neuroinflammation contributes to central sensitization progression [2–4]. Given that neurons in superficial dorsal horn of ApoE4-TR mice exhibited higher excitability, we compared the inflammatory response in ApoE4-TR and ApoE3-TR mice. Specifically, the expression of key inflammatory cytokines in the spinal dorsal horn was assessed using the ELISA at 14 days post-SNI (Fig. 3A). According to the results, IL-6, IL-1β, and TNF-α were all elevated after nerve surgery. Compared to ApoE3-TR mice, ApoE4-TR mice showed significantly higher levels of these inflammatory cytokines (Fig. 3B). Additionally, spinal dorsal horn tissues were subjected to RT-qPCR analysis, which also revealed higher expression of IL-6, IL-1β, and TNF-α in ApoE4-TR mice (Fig. 3C).

To further confirm the differential inflammatory response between the two ApoE genotypes, astrocytes were isolated from the spinal cords of neonatal ApoE3-TR and ApoE4-TR mice. Cultured cells were subjected to immunofluorescence staining with an anti-GFAP antibody, and over 98% were identified as GFAP^+^ astrocytes in both groups (Fig. 3D, E). To mimic an inflammatory response in vitro, ApoE3 and ApoE4 astrocytes were treated with a cytokine mixture (CYM) consisting of IL-1β, TNF-α, and IFN-γ [26], which are known to induce a pro-inflammatory environment in astrocytes associated with neuropathic pain [27–30]. IL-6, IL-1β, and TNF-α were detected because they are key inflammatory cytokines that could be released by astrocytes and contribute to central sensitization [2, 31–34] (Fig. 3F). After 6 h of treatment, the culture medium was replaced with fresh medium. To verify that residual cytokines had been completely removed, ELISA was performed 3 min after the medium change. The results showed very low and comparable levels of the three cytokines, confirming that the washing was effective (Fig. 3G). Then, separate samples were collected 12 h later to evaluate cytokine release over time. Compared to ApoE3 astrocytes, ApoE4 astrocytes released higher levels of IL-6, IL-1β, and TNF-α into the culture medium (Fig. 3G). Additionally, cytokine gene expression was assessed using RT-qPCR, confirming a greater upregulation of inflammatory genes in ApoE4 astrocytes (Fig. 3H). These findings collectively suggested that, under pathological conditions, ApoE4 was correlated with higher levels of inflammatory cytokines both in vivo and in vitro.

### Reduced spermidine in ApoE4-TR mice following nerve injury

**Fig. 4:**
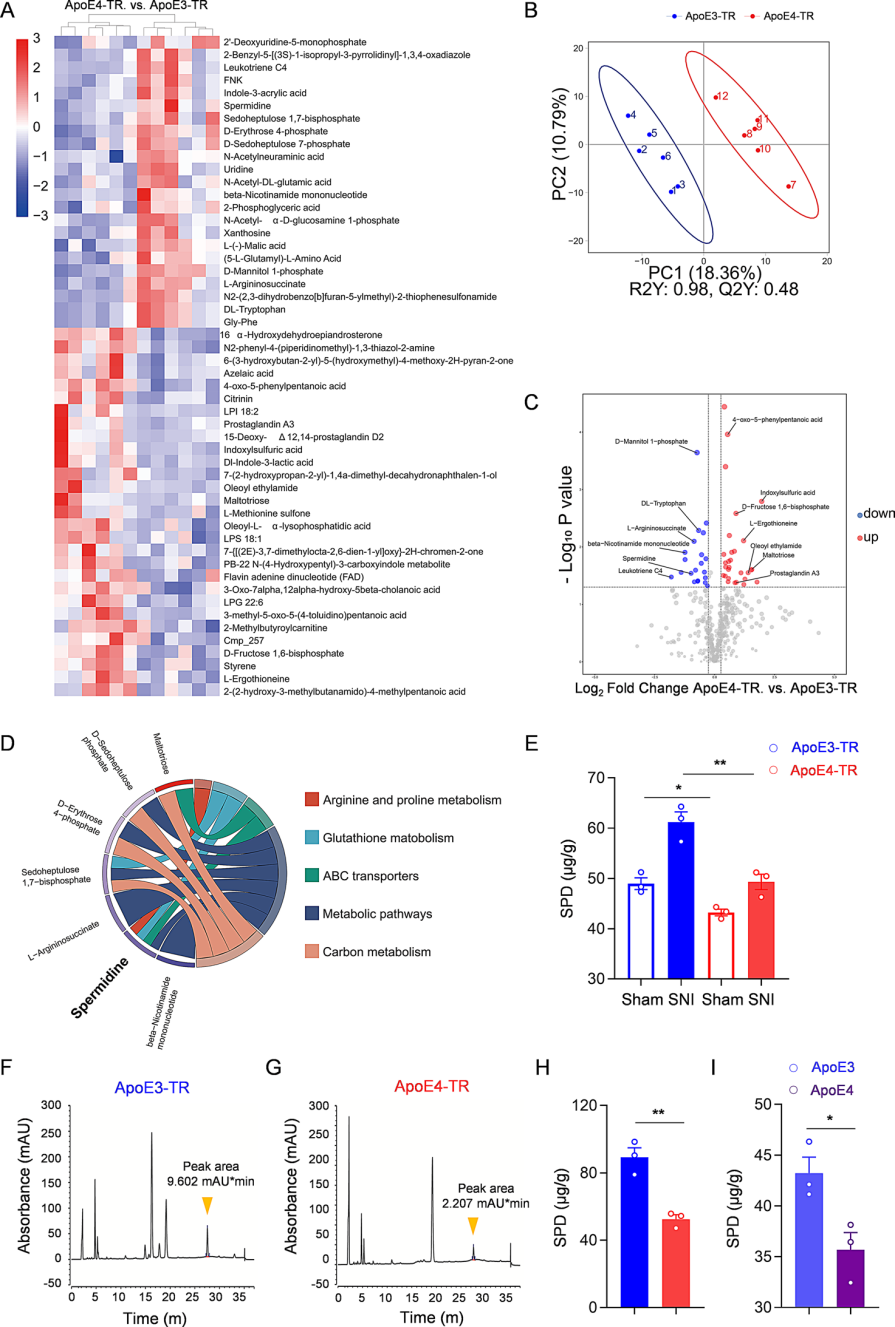
Reduced spermidine in ApoE4-TR mice following nerve injury. **(A)** Heatmap displaying the relative abundance of metabolites in the dorsal horn of the spinal cord in ApoE3-TR and ApoE4-TR mice 14 days after SNI. **(B)** PLS-DA of metabolites was conducted in ApoE3-TR and ApoE4-TR mice measured by both electrospray ionization in positive mode (ESI^+^) and negative mode (ESI^−^). **(C)** The Volcano plot of spinal dorsal horn metabolomics comparing ApoE3-TR and ApoE4-TR mice. **(D)** KEGG chord plot showing the relationship between the selected pathways and their corresponding metabolites. **(E)** ELISA measurement of spermidine levels in ApoE3-TR and ApoE4-TR mice before and 14 days after the SNI surgery. *n* = 3 biologically independent samples. **(F**,** G)** Representative HPLC chromatograms showing the levels of spermidine in the spinal dorsal horn of ApoE3-TR **(F)** and ApoE4-TR **(G)** mice after SNI. The peak area indicated by yellow arrowheads corresponds to spermidine. The x-axis represents retention time (min), while the y-axis represents absorbance (mAU). **(H)** Quantification of spermidine levels in ApoE3-TR and ApoE4-TR mice after the SNI. *n* = 3 biologically independent samples. **(I)** ELISA measurement of spermidine levels in primary astrocytes from ApoE3 and ApoE4 mice. *n* = 3 biologically independent samples. All data are expressed as mean ± SEM. Statistical comparisons were conducted with one-way ANOVA followed by Tukey’s post hoc test **(E)**; and unpaired Student’s t-test **(H, I)**. *P < 0.05, and **P < 0.01

Given that ApoE is a metabolic regulator in humans and mice, we further sought to investigate whether variations in inflammatory cytokines correlate with metabolic differences. The spinal dorsal horns from the injured side of ApoE3-TR and ApoE4-TR mice were extracted for metabolomic analysis at 14 days. As expected, the ApoE3-TR and ApoE4-TR groups exhibited distinct metabolites, as determined by untargeted metabolomics using UHPLC-MS/MS followed by multivariate statistical analysis (Fig. 4A). Moreover, partial least squares discriminant analysis (PLS-DA) of metabolomic data revealed a notable separation between the two groups in the two-dimensional score plot, implying significant metabolic differences. The model’s R²Y value was 0.98, indicating high goodness of fit, while its Q²Y value was 0.48, demonstrating good predictive capacity (Fig. 4B). Differential metabolite analysis between the two groups found 24 downregulated and 29 upregulated metabolites in ApoE4-TR mice under combined positive and negative ion modes (Fig. 4C). Moreover, KEGG enrichment analysis of the differentially expressed metabolites identified enrichment in several pathways, including arginine and proline metabolism, glutathione metabolism, metabolic pathways, carbon metabolism and ABC transporters. These pathways were subsequently visualized using chord diagram analysis. Notably, spermidine was found to be a key metabolite implicated in multiple enriched pathways (Fig. 4D).

Spermidine is primarily stored in astrocytes [35–38], which is also the main cellular location of ApoE in CNS following SNI. Additionally, spermidine plays a significant role in critical cellular processes, such as regulating autophagy, promoting cellular stress resistance, and modulating mitochondrial function, all of which are essential for the maintenance of astrocyte function and the regulation of inflammatory responses. Therefore, we assessed whether the levels of spermidine varied according to ApoE genotypes. Interestingly, spermidine was slightly lower in ApoE4-TR mice than in ApoE3-TR mice under physiological conditions. At 14 days post-SNI, both groups exhibited increased spermidine levels in the spinal dorsal horn. However, the increase of spermidine in ApoE4-TR mice was significantly lower than in ApoE3-TR mice (Fig. 4E). Spermidine levels were further examined using HPLC (Fig. 4F, G). Again, ApoE4-TR mice exhibited lower spermidine levels (52.48 ± 2.66 µg/g) compared to ApoE3-TR mice (89.26 ± 5.65 µg/g) post-SNI (Fig. 4H). We also quantified the spermidine content in CYM-treated ApoE3 and ApoE4 astrocytes in vitro using ELISA. Results showed a concentration of 35.69 ± 1.67 µg/g in the ApoE4 group, and 43.21 ± 1.59 µg/g in the ApoE3 group (Fig. 4I), aligning with the in vivo findings. Together, these results indicated that, under these pathological conditions, the levels of spermidine in ApoE4-TR mice were further decreased compared to ApoE3-TR mice.

### Requirement of high-dose spermidine for pain relief in ApoE4-TR mice due to limited spermidine retention

**Fig. 5:**
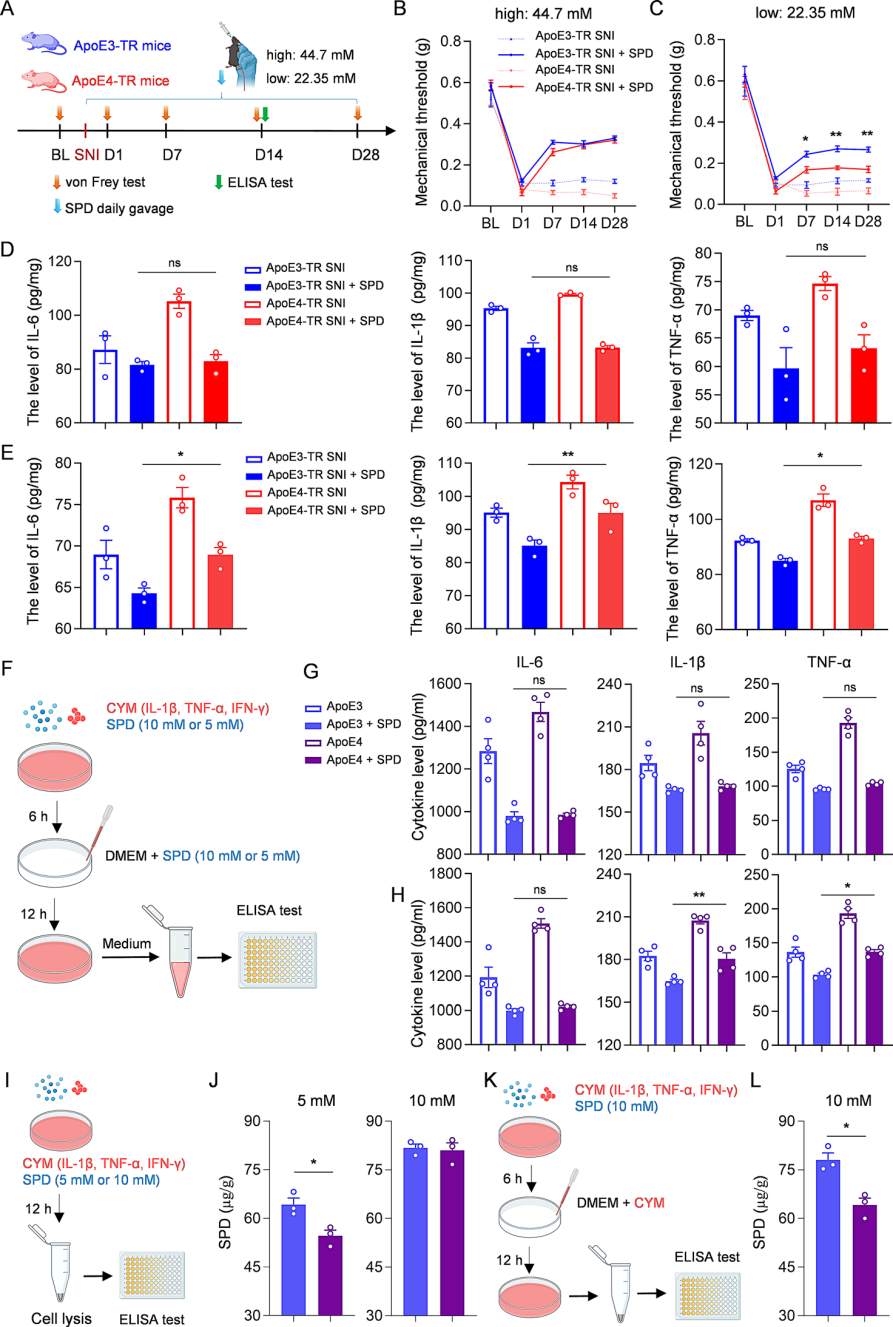
Requirement of high-dose spermidine for pain relief in ApoE4-TR mice due to limited spermidine retention. **(A)** Schematic of experimental design and spermidine treatment (200 µl 44.7 mM or 22.35 mM). **(B**,** C)** Mechanical thresholds of ApoE3-TR and ApoE4-TR mice before and after SNI, with or without spermidine gavage treatment at high dose **(B)** or low dose **(C)**. *n* = 10 mice per group. Cohen’s d for ApoE3-TR vs. ApoE4-TR post-SNI following 22.35 mM SPD gavage: 2.29 (D7), 3.58 (D14), and 3.09 (D28). **(D**,** E)** ELISA measurement of pro-inflammatory cytokine levels in the spinal dorsal horn of ApoE3-TR and ApoE4-TR mice 14 days after 44.7 mM spermidine **(D)** or 22.35 mM spermidine **(E)** treatment. *n* = 3 biologically independent samples. **(F)** Experimental scheme of CYM and spermidine treatment. **(G)** ELISA detection of pro-inflammatory cytokine levels in the supernatant of ApoE3 and ApoE4 astrocytes with or without 10 mM spermidine treatment. *n* = 3 biologically independent samples. **(H)** ELISA detection of pro-inflammatory cytokine levels in the supernatant of ApoE3 and ApoE4 astrocytes with or without 5 mM spermidine treatment. *n* = 3 biologically independent samples. **(I)** An experimental scheme for the detection of intracellular spermidine content. **(J)** ELISA analysis of the spermidine levels in the ApoE3 and ApoE4 astrocytes after treatment with 5 mM (right) or 10 mM (left) spermidine. *n* = 3 independent biological samples. **(K)** Experimental scheme for the determination of spermidine retention capacity in astrocytes. **(L)** ELISA analysis of intracellular spermidine levels in ApoE3 and ApoE4 astrocytes after 10 mM spermidine treatment and subsequent medium replacement. *n* = 3 independent biological samples. All data are expressed as mean ± SEM. Statistical comparisons were conducted with two-way ANOVA followed by Bonferroni’s post hoc test **(B, C)**; one-way ANOVA followed by Tukey’s post hoc test **(D, E, G, H)**; and unpaired Student’s t-test **(J, L)**. *P < 0.05, and **P < 0.01

To determine whether the increased pain sensitivity in ApoE4-TR mice was associated with reduced spermidine levels, two doses of spermidine (44.7 mM or 22.35 mM) were administered daily via oral gavage to ApoE3-TR and ApoE4-TR mice, starting from day 1 post-SNI (Fig. 5A), based on previous literature [39, 40]. Mechanical pain thresholds were assessed on days 7, 14, and 28. From day 7, both ApoE3-TR and ApoE4-TR mice exhibited similar levels of pain relief in high dose groups (Fig. 5B). However, in the low-dose groups, although both genotypes exhibited increased mechanical thresholds as early as day 7, ApoE4-TR mice exhibited significantly lower thresholds (Fig. 5C). After 14 days of spermidine treatment, spinal dorsal horn tissues were subjected to ELISA analysis. Although IL-6, IL-1β, and TNF-α levels were higher in ApoE4-TR mice, high-dose spermidine treatment reduced these inflammatory cytokines to comparable levels in both genotypes, consistent with the mechanical pain behaviors (Fig. 5D). In contrast, in the low-dose groups, though inflammatory cytokine levels were generally decreased in ApoE3-TR mice and ApoE4-TR mice, IL-6, IL-1β, and TNF-α remained higher in ApoE4-TR mice than in ApoE3-TR mice (Fig. 5E). Additionally, primary astrocytes isolated from ApoE3-TR and ApoE4-TR mice were examined for their response to CYM stimulation, with or without spermidine treatment. Consistent with the in vivo results, IL-6, IL-1β, and TNF-α were all reduced to comparable levels in the high-dose (10 mM) groups (Fig. 5F, G). In contrast, treatment with 5 mM spermidine led to a smaller reduction in these cytokines, with higher levels of IL-6 and IL-1β observed in ApoE4 astrocytes compared to ApoE3 astrocytes (Fig. 5H). These results suggested that, compared to ApoE3-TR mice, more spermidine was required to alleviate neuropathic pain in ApoE4-TR mice.

Since astrocytes are the main storage sites for spermidine in the CNS [35–38], we examined whether their retention capacity affected spermidine’s pain-relieving effects. After 12 h of exposure to 5 mM spermidine and CYM treatment, ApoE3 astrocytes showed significantly higher intracellular spermidine levels than ApoE4 astrocytes, as determined by ELISA (Fig. 5I, J). However, contrary to the results from the low-dosage treatment, after 12 h treatment with 10 mM spermidine and CYM, no differences were observed between the two groups (Fig. 5J). Therefore, we hypothesized that under high-dose spermidine conditions, ApoE4 astrocytes may continuously uptake spermidine from the extracellular medium, resulting in intracellular spermidine levels comparable to those in ApoE3 astrocytes. To test this hypothesis, spermidine was removed from the culture medium after 6 h of high-dose (10 mM) treatment, and astrocytes were subjected to CYM stimulation alone for an additional 12 h (Fig. 5K). Then, the intracellular spermidine level was assessed and the results revealed that spermidine levels in ApoE4 astrocytes have become much lower than those in ApoE3 astrocytes, thus confirming our hypothesis (Fig. 5L). These findings collectively indicate that ApoE4 astrocytes exhibit reduced spermidine retention capacity compared to ApoE3 astrocytes.

### *Nos2* was upregulated in ApoE4-TR mice

**Fig. 6:**
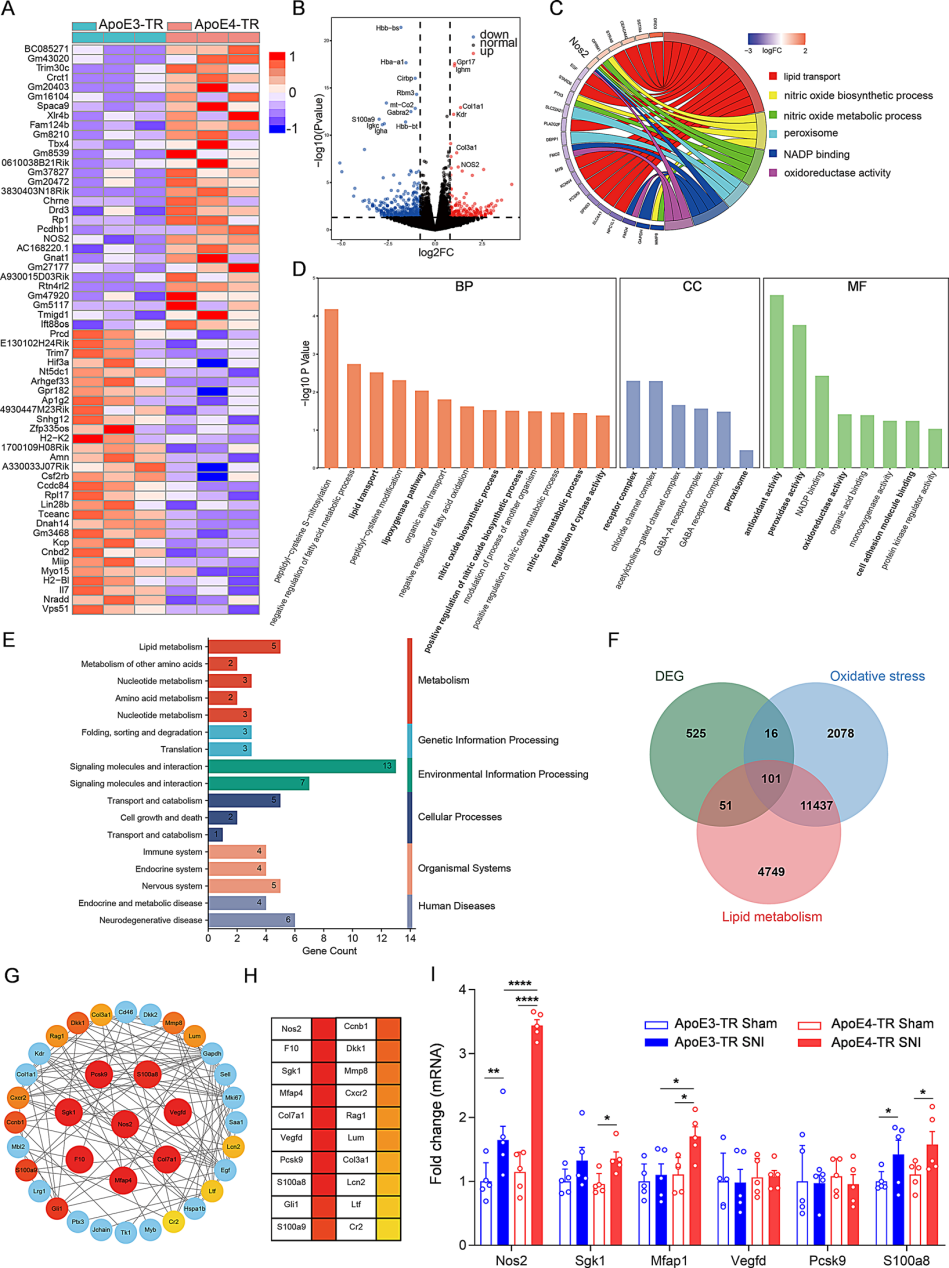
*Nos2* was upregulated in ApoE4-TR mice. **(A)** Heatmap showing the changes in gene expression of ApoE4-TR mice compared to ApoE3-TR mice after SNI. Each row represents a gene, and each column represents a sample. Red indicates higher expression levels, while blue indicates lower expression levels. **(B)** The volcano plot shows significantly altered genes in ApoE4-TR mice after SNI. Blue dots represent downregulated genes, red dots represent upregulated genes, and black dots represent genes with no significant changes. **(C)** The GO chord plot displays the relationship between the selected pathways and their corresponding genes. **(D)** Significantly enriched GO items in biological processes (BP), cellular components (CC), and molecular functions (MF). **(E)** Bar chart showing the KEGG pathways analysis for differentially expressed genes. **(F)** The Venn diagram shows the shared and unique genes among the three groups of differentially expressed genes, lipid metabolism gene set, and oxidative stress gene set. **(G)** Illustration of the clusters in the PPI network. **(H)** The top 20 genes ranked after filtering the PPI interaction network. **(I)** RT-qPCR analysis of the top six gene expressions in the dorsal horn of ApoE3-TR and ApoE4-TR mice 14 days after SNI, including sham-operated controls. Data were normalized to the ApoE3-TR Sham group and presented as “fold change” in the graphs. *n* = 5 independent biological samples. Gene expression was analyzed using a one-way ANOVA followed by Tukey’s post hoc test. *P < 0.05, **P < 0.01 and ****P < 0.0001

Spermidine, as a metabolite, was found to influence many downstream gene transcription factors, such as Runx2, Osx, ALP, and ATF2 [39, 41–43]. In this regard, we sought to address how spermidine modulates the differences in pain sensitivity observed between ApoE3-TR and ApoE4-TR mice through relevant genes. Spinal dorsal horn tissues of ApoE3-TR and ApoE4-TR mice at 14 days post-SNI were subjected to transcriptome sequencing (Fig. 6A). The analysis yielded 693 differentially expressed genes (DEGs, *P* < 0.05 and |fold change| ≥ 1) between ApoE3-TR and ApoE4-TR mice, of which 212 were upregulated and 481 downregulated, respectively (Fig. 6B). The DEGs in ApoE4-TR mice were more involved in the regulation of neuropathic pain-associated biological processes (BP), including lipid transport, the lipoxygenase pathway, positive nitric oxide biosynthesis regulation, and cyclase activity regulation. Regarding cellular components (CC), ApoE4 primarily affected receptor complexes and peroxidases, while molecular functions (MF) influenced by ApoE4 included peroxidase activity, oxidoreductase activity, and antioxidant activity (Fig. 6C-E).

Biological enrichment analysis suggests a close relationship with oxidative stress and lipid metabolism. Therefore, we propose that, in addition to differential gene expression, the key genes are likely involved in oxidative stress and lipid metabolism processes. To identify the key DEGs, oxidative stress- and lipid metabolism-related genes were downloaded from the GeneCards database and intersected with the 693 DEGs identified herein, yielding 101 key gene set (Fig. 6F). These 101 DEGs were then uploaded to the STRING database using a minimum interaction score of “medium confidence (0.400)” for screening. Protein-protein interaction (PPI) networks were visualized using Cytoscape-CytoHubba. Based on degree clustering coefficients, the most stable and highly ranked genes within the network were those with numerous nodes and edges (Fig. 6G). As shown, the top eight genes were *Nos2*, *F10*, *Sgk1*, *Mfap4*, *Col7a1*, *Vegfd*, *Pcsk9*, and *S100a8* (Fig. 6H). However, *F10* and *Col7a1* were barely associated with astrocytes. The relative expression levels of *Nos2*, *Sgk1*, *Mfap4*, *Vegfd*, *Pcsk9*, and *S100a8* in the spinal dorsal horn of ApoE3-TR and ApoE4-TR mice under sham or SNI conditions were further evaluated by RT-qPCR. These genes showed comparable expression levels between the two genotypes under sham conditions. However, following SNI, *Nos2* and *Mfap1* were significantly upregulated in ApoE4-TR mice compared to ApoE3-TR mice, with *Nos2* showing a more pronounced increase. (Fig. 6I).

### Spermidine suppressed NOS2 expression via the NF-κB pathway

**Fig. 7:**
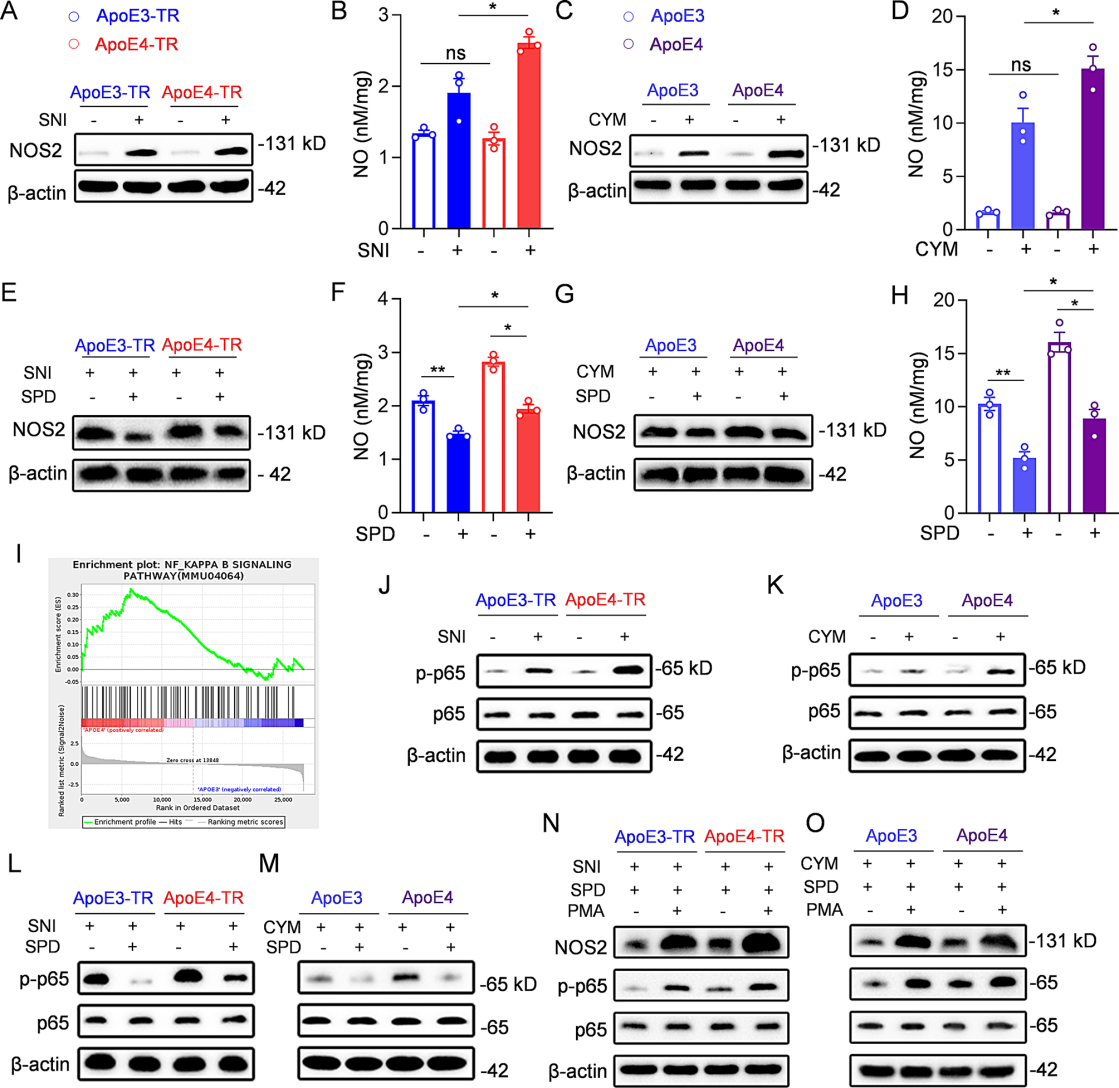
Spermidine reduced NOS2 expression via the NF-κB pathway. **(A)** Western blot analysis of the NOS2 expression in the spinal dorsal horn of ApoE3-TR and ApoE4-TR mice in sham or SNI group, with β-actin serving as the loading control. **(B)** NO levels in the spinal dorsal horn of ApoE3-TR and ApoE4-TR mice in sham or SNI group. **(C)** Western blot analysis of intracellular NOS2 expressions in ApoE3 and ApoE4 astrocytes with or without CYM stimulation. **(D)** NO levels in ApoE3 and ApoE4 astrocytes with or without CYM stimulation. **(E)** Western blot analysis of NOS2 expression in the spinal dorsal horn receiving low-dose (22.35 mM) spermidine or PBS treatment in ApoE3-TR and ApoE4-TR mice following SNI. **(F)** The content of NO in the spinal dorsal horn of ApoE3-TR and ApoE4-TR mice receiving spermidine gavage or PBS treatment after SNI. **(G)** Western blot analysis of intracellular NOS2 expression in ApoE3 and ApoE4 astrocytes following CYM stimulation with or without spermidine (5 mM) treatment in mice. **(H)** NO levels in ApoE3 and ApoE4 astrocytes with or without spermidine treatment after CYM stimulation. **(I)** The NF-κB pathway was enriched by GSEA in ApoE4-TR mice after SNI injury. **(J)** Western blot analysis of p65 and p-p65 expression in the spinal dorsal horn of ApoE3-TR and ApoE4-TR mice in sham or SNI group. **(K)** Western blot analysis of intracellular p65 and p-p65 expression in ApoE3 and ApoE4 astrocytes with or without CYM stimulation. **(L)** Western blot analysis of p65 and p-p65 expression in the spinal dorsal horn of ApoE3-TR and ApoE4-TR mice after SNI, with or without spermidine (22.35 mM) treatment. **(M)** Western blot analysis of p65 and p-p65 expression in ApoE3 and ApoE4 astrocytes after CYM stimulation, with or without spermidine (5 mM) treatment. **(N)** Western blot analysis of NOS2, p65 and p-p65 expression in the spinal dorsal horn of ApoE3-TR and ApoE4-TR mice after SNI and spermidine gavage treatment, with or without intrathecal PMA injection. **(O)** Western blot analysis of NOS2, p65 and p-p65 expression in ApoE3 and ApoE4 astrocytes after the treatment with CYM and spermidine, with or without PMA addition. *n* = 3 independent biological replicates per group for all Western blot and NO analysis. All data are expressed as mean ± SEM. Statistical comparisons were conducted with one-way ANOVA followed by Tukey’s post hoc test **(B, D, F, H)**. *P < 0.05, and **P < 0.01

*Nos2* encodes inducible nitric oxide synthase (iNOS/NOS2), an enzyme upregulated in astrocytes under inflammatory and pathological conditions, responsible for producing nitric oxide (NO) [44–46] and developing hyperalgesia. Therefore, we further determined whether the change of *Nos2* mRNA in ApoE4-TR mice was reflected at the protein level. Both transgenic mice were subjected to Western blot analysis post-SNI, consistent with the RT-qPCR results, ApoE4-TR mice exhibited significant upregulation of NOS2 (Fig. 7A, S4A). Notably, the NO levels in the spinal dorsal horn were also increased in ApoE4-TR group (Fig. 7B). Furthermore, consistent with the in vivo findings, treatment of isolated astrocytes with CYM led to increased expression of NOS2 and NO in ApoE4 astrocytes (Fig. 7C, D, S4B). Given that spermidine can regulate downstream gene expression and *Nos2* was the main altered gene, we further checked whether spermidine regulated *Nos2* in ApoE3/4-TR mice. After two weeks of spermidine treatment (22.35 mM), Western blot analysis of the spinal dorsal horn in SNI mice revealed that the injury-induced upregulation of NOS2 was significantly reversed (Fig. 7E, S4C), a phenomenon also observed in CYM-treated astrocytes. Moreover, NO levels were decreased following spermidine (5 mM) treatment both in vivo and in vitro (Fig. 7F-H, S4D).

To elucidate the mechanism by which spermidine regulates NOS2, we performed gene set enrichment analysis (GSEA) to identify key pathways associated with ApoE4, which revealed ApoE4 significantly correlated with the NF-κB signaling pathway (Fig. 7I). Consequently, we examined the NF-κB pathway in ApoE3/4-TR mice. After SNI, ApoE4- and ApoE3-TR mice exhibited comparable p65 levels. However, compared to ApoE3-TR mice, ApoE4-TR mice showed significantly higher levels of phosphorylated p65 (p-p65) (Fig. 7J, S4E, F). Similar results were observed in CYM-stimulated primary astrocytes in vitro (Fig. 7K, S4G, H). Interestingly, spermidine treatment at low-dose (22.35 mM) downregulated p-p65 in both transgenic mice groups, although p-p65 levels in ApoE4-TR mice remained higher than those in ApoE3-TR mice (Fig. 7L, S4I, J). This finding was further confirmed in vitro from primary astrocytes (Fig. 7M, S4K, L). Western blot revealed no significant differences in the expression of NOS2, p65 and p-p65 between spermidine-treated and untreated sham groups (Fig. S4M, N), indicating that spermidine does not affect NOS2 or NF-κB activity under non-neuropathic conditions. Finally, to investigate whether spermidine regulates NOS2 expression through the NF-κB pathway, phorbol 12-myristate 13-acetate (PMA), an NF-κB activator, was intrathecally injected into spermidine-treated mice. Following PMA administration, the spermidine-induced downregulation of p-p65 was significantly reversed, accompanied by an increase in NOS2 levels (Fig. 7N, S4O-Q). Similarly, in astrocytes pre-treated with spermidine, PMA treatment led to a concurrent upregulation of p-p65 and NOS2 levels in vitro (Fig. 7O, S4R-T). These findings suggested that spermidine regulated NOS2 through the NF-κB pathway, contributing to the increased mechanical hypersensitivity observed in ApoE4-TR mice.

## Discussion

**Fig. 8:**
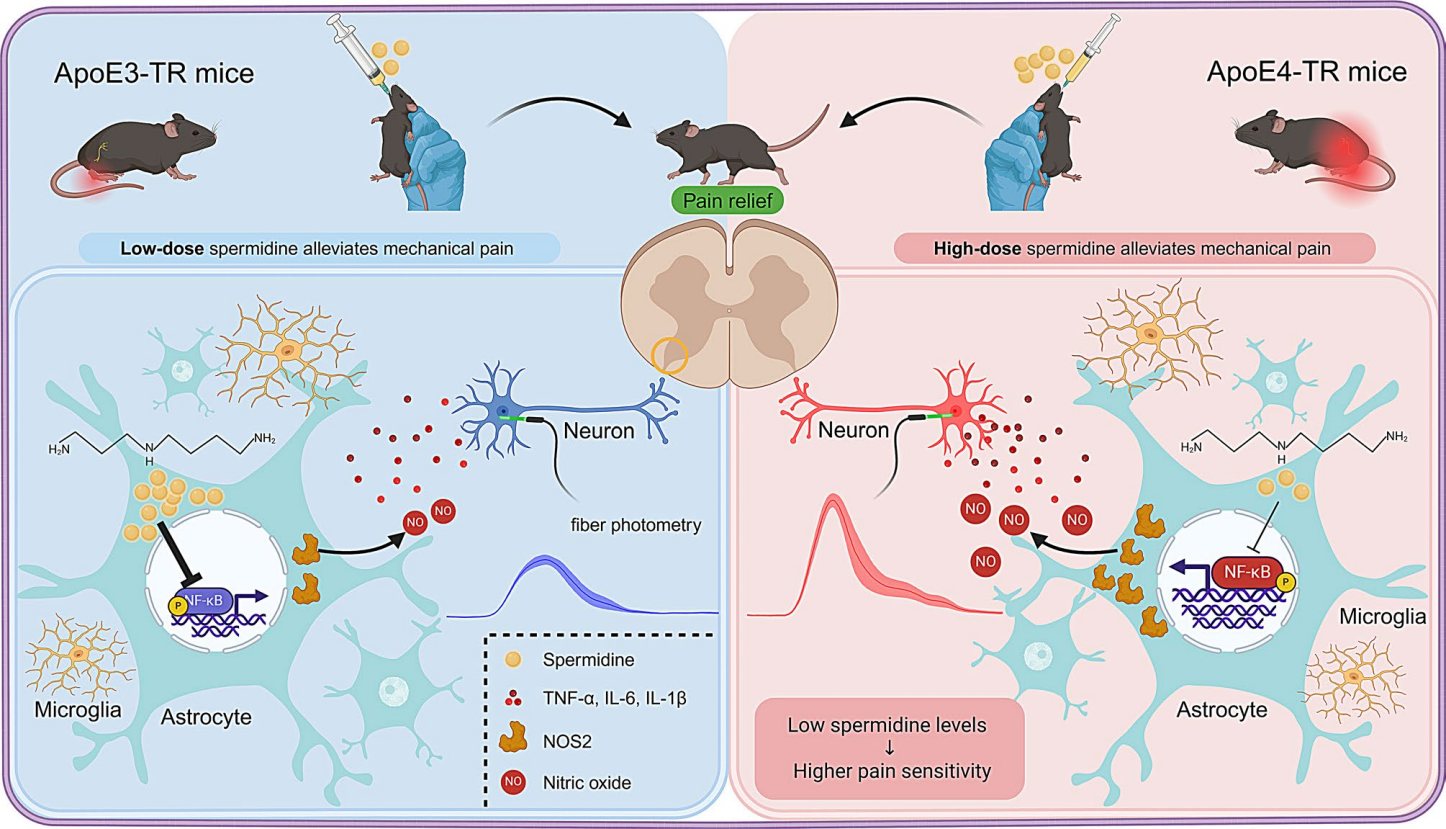
Astrocytic spermidine insufficiency contributes to enhanced pain sensitivity associated with ApoE4 through the NF-κB/NOS2 pathway

In this study, we demonstrated that ApoE4-TR mice exhibited enhanced pain sensitivity compared to ApoE3-TR mice following peripheral nerve injury. Two weeks after sciatic nerve injury, ApoE4-TR mice showed reduced mechanical thresholds, increased neuronal excitability in the superficial dorsal horn, and elevated levels of pro-inflammatory cytokines. Metabolomic profiling identified spermidine as a significant differentially expressed metabolite in the dorsal horn, a finding validated by ELISA and HPLC. Spermidine administration attenuated neuropathic pain in a dose-dependent manner following SNI. Notably, higher doses of spermidine were required to achieve pain relief in ApoE4-TR mice, likely due to limited spermidine retention in astrocytes. Transcriptomic analysis demonstrated that *Nos2* was a critical differentially expressed gene, and GSEA suggested the NF-κB pathway as a potential key regulator. Mechanistic studies further demonstrated that spermidine treatment inhibited NOS2 via the NF-κB pathway both in vivo and in vitro, thereby contributing to the alleviation of mechanical hypersensitivity (Fig. 8).

ApoE4 is the most important genetic risk factor for late-onset Alzheimer’s disease [8, 10, 47]. Besides, ApoE4 also correlates with increased diabetic neuropathy severity [15, 16]. However, whether ApoE4 mice exhibit different pain sensitivity compared to other ApoE isoforms after nerve injury remains unclear. ApoE is a well-established multifunctional regulator of neuroinflammation. In the brain, ApoE4-expressing microglia and astrocytes contribute to AD progression [48–50]. In our study, ApoE was predominantly colocalized with astrocytes, and the proportion of ApoE-positive astrocytes further increased 14 days after SNI (Fig. 1J). Studies have shown that ApoE negatively regulates inflammatory pain [18]. Whereas, following nerve injury, no significant differences were observed in the proliferation of astrocytes or microglia, nor in ApoE protein levels between ApoE3-TR and ApoE4-TR mice. Notably, under normal physiological conditions, ApoE3-TR and ApoE4-TR mice exhibited comparable mechanical pain thresholds. Post-SNI, however, ApoE4-TR mice displayed reduced mechanical pain thresholds and elevated calcium imaging signals, indicative of heightened neuronal excitability in the dorsal horn (Fig. 1G-I).

Recent studies utilizing large cohort data from the UK Biobank revealed that male ApoE4 carriers exhibit a reduced risk of chronic back pain and headaches [17]. In the present study, we found that ApoE4-TR mice displayed increased mechanical pain sensitivity in the SNI model. This discrepancy may arise from the possibility that mechanisms and pathogenesis of back pain and headaches may differ from those of post-injury neuropathic pain. Additionally, data in the UK Biobank did not encompass pain outcomes following peripheral nerve injury [17]. Considering the existence of reports that ApoE was also detected in spinal microglia or neurons [50–52], we employed LSL-hApoE3/4-3xflag mice and used AAV-mediated GFAP-Cre delivery to drive hApoE3/4 expression in dorsal horn astrocytes. The results indicated that LSL-ApoE4 mice exhibited greater mechanical hypersensitivity compared to LSL-ApoE3 mice, suggesting that ApoE4 enhances pain sensitivity primarily through astrocyte modulation (Fig. 2).

Astrocytes account for 20–40% of all glial cells in the central nervous system [53]. Numerous studies have discovered that the activation of astrocytes and microglia triggers neuroinflammation, enhancing the development and maintenance of neuropathic pain following nerve injury [54–56]. Research has shown that ApoE regulates pain by modulating the proliferation of astrocytes [18]. In this study, we found that increased release of inflammatory factors by astrocytes, rather than differential glial cell activation, contributed to the differences in pain sensitivity between ApoE3-TR and ApoE4-TR mice. Higher levels of inflammatory factors reflect more severe central sensitization. Although ApoE may negatively regulate the occurrence of pain in inflammatory contexts, astrocytes in ApoE4 mice may also aggravate pain severity due to metabolic disturbances [57–59].

Spermidine is a naturally occurring polyamine which regulates various cellular processes, including autophagy and oxidative stress, and has recently been investigated for its anti-aging effects [60, 61]. Existing research indicates that spermidine is primarily retained and accumulated in astrocytes within the central nervous system, where it prevents oxidative stress [62, 63]. Previous studies have shown that spermidine levels increase in the dorsal root ganglia following sciatic nerve injury, with sustained upregulation between 2 and 7 days post-injury, reflecting a dynamic but overall elevated response to peripheral nerve damage [64]. Consistent with these findings, we observed increased spermidine levels in the spinal dorsal horn of both ApoE3-TR and ApoE4-TR mice 14 days after SNI (Fig. 4E-G), indicating that spermidine upregulation occurs following nerve injury, regardless of the presence of pain behaviors.

Spermidine has been shown to exhibit neuroprotective effects. In a rat model of optic nerve crush, a single intravitreal injection of spermidine significantly promoted optic nerve regeneration, highlighting its therapeutic effect on neural repair [64]. Additionally, recent evidence has shown that spermidine can alleviate neuropathic pain by reducing oxidative stress levels in a chronic constriction injury rat model [65]. Despite a baseline difference in spermidine levels between ApoE3-TR and ApoE4-TR mice in the spinal cord prior to SNI surgery, this physiological disparity did not result in variations in the baseline mechanical pain response (Figs. 4E and 5B and C). This may be attributed to the fact that under normal conditions, the retained spermidine in astrocytes does not require activation of its protective mechanism. After SNI, ApoE4-TR mice exhibited lower spermidine levels compared to ApoE3-TR mice, indicating a reduced protective capacity that may contribute to their heightened pain sensitivity. However, the precise mechanisms underlying neuropathic pain remain to be fully elucidated. Following high-dose spermidine treatment in vivo and in vitro, neuropathic pain was similarly attenuated in both ApoE3-TR and ApoE4-TR mice (Fig. 5B). The differences in pain sensitivity between the two genotypes were eliminated post-treatment, with mechanical pain thresholds and inflammatory factors converging to comparable levels. Notably, after administering a low dose of spermidine, ApoE4 mice exhibited persistently lower thresholds (Fig. 5C), emphasizing the heterogeneity in disease progression and treatment response between ApoE3- and ApoE4-TR mice.

Hepatocytes are the primary source of ApoE in peripheral circulation and are also capable of producing inflammatory cytokines [66, 67]. Although SNI surgery primarily affects the sciatic nerve and induces central sensitization in the spinal dorsal horn without directly damaging hepatocytes, their role in inflammatory regulation cannot be overlooked. Spermidine may alleviate pain indirectly by reducing oxidative stress and modulating inflammatory responses, thereby influencing cytokine production in hepatocytes. However, since this study focused primarily on the direct effects of spermidine within the spinal cord, its potential impact on hepatocytes requires further investigation for validation.

We further determined whether the differences in astrocytic spermidine retention capacity between ApoE genotypes could persist after spermidine administration. The levels of astrocytic spermidine after low-dose treatment confirmed that ApoE3 and ApoE4 astrocytes had differential effects on the retention of spermidine. Moreover, after high-dose spermidine supplementation, we investigated whether spermidine could be eliminated from the culture medium. Our results suggest that, despite their decreased retention capacity, ApoE4 astrocytes can continuously uptake spermidine from the medium at high concentrations (Fig. 5I-L), thereby exerting protective effects.

Numerous studies have investigated the impact of ApoE genotype on AD mouse models, human brains, and iPSC-derived astrocytes, indicating that ApoE4 contributes to the occurrence of pro-inflammatory responses in transcriptomic analyses [68–70]. In this study, we performed transcriptome sequencing to identify differentially expressed genes in ApoE3-TR and ApoE4-TR mice after SNI surgery. GO analysis showed that ApoE4-related differentially expressed genes were enriched in pathways related to lipid transport, lipoxygenase, nitric oxide biosynthesis, peroxidase activity, and redox activity (Fig. 6D). These findings indicated that astrocytes in the spinal cord had similar differential responses as observed in the brain. Evidence from prior investigations have demonstrated that spermidine can regulate NOS2 mRNA expression in vitro [71, 72]. Given the differences in spermidine levels between ApoE3 and ApoE4, and the identification of *Nos2* as a significant differentially expressed gene in our transcriptomic analysis, we further investigated whether spermidine regulated the NOS2 expression. The results indicated that spermidine negatively regulated NOS2 both in vivo and in vitro, which contributed to the differences in pain thresholds. Moreover, GSEA analysis of the transcriptome showed that the NF-κB pathway was significantly enriched in ApoE4-TR mice. Notably, treatment with an NF-κB agonist abolished the spermidine-induced downregulation of p-p65 (Fig. 7N, O), suggesting that spermidine exerts its analgesic effects, at least in part, through inhibition of the NF-κB signaling pathway. Although previous studies have demonstrated that spermidine can regulate the NF-κB/p65 axis [73], our findings provide additional evidence for this mechanism specifically in the context of neuropathic pain. More importantly, our data reveal a genotype-specific effect, as the regulatory impact of spermidine on NF-κB signaling was particularly pronounced in ApoE4-TR mice.

Despite the significant advances made in this study, several limitations remain to be addressed in future research. First, inflammatory cytokine levels in the spinal dorsal horn were only assessed at 14 days post-SNI, during which ApoE4-TR mice exhibited significantly higher levels of IL-6, IL-1β, and TNF-α compared to ApoE3-TR mice. While these results suggest a heightened inflammatory response in ApoE4-TR mice, extended observation periods and larger sample size are needed to determine whether this elevation persists and contributes to the long-term maintenance of neuropathic pain. Second, the transcriptomic analysis in this study compared ApoE3-TR and ApoE4-TR mice only after nerve injury, without including sham-operated controls. As such, it cannot be fully ruled out that some of the observed gene expression differences may reflect inherent genotype-specific variations rather than changes specifically related to neuropathic pain. The inclusion of sham-operated controls in future transcriptomic analyses would provide greater clarity in distinguishing baseline genotype effects from pain-induced transcriptional alterations. Lastly, previous studies have shown that spermidine levels decline with age, and its effectiveness in modulating neuropathic pain may vary across different age groups. As this study focused on young adult mice (8–10 weeks old), future research involving aged mice will be important for further clarifying the age-related effects of spermidine in neuropathic pain treatment and for optimizing its therapeutic dosing across different age groups and clinical conditions.

This study demonstrates that neuropathic pain exhibits distinct phenotypes depending on ApoE genotypes. We have characterized the differential responses of primary ApoE3 and ApoE4 astrocytes to inflammatory stimuli in the spinal cord and investigated the underlying metabolic and transcriptomic mechanisms. Importantly, these findings highlight spermidine as a potential therapeutic agent for neuropathic pain, with treatment strategies requiring customization based on ApoE genotype to optimize efficacy.

## Electronic supplementary material

Below is the link to the electronic supplementary material.

Supplementary Material 1

Supplementary Material 2

## Data Availability

No datasets were generated or analysed during the current study.

## References

[1] Campbell JN, Meyer RA (2006) Mechanisms of neuropathic pain. Neuron 52(1):77–92. 10.1016/j.neuron.2006.09.021 17015228 10.1016/j.neuron.2006.09.021 PMC1810425

[2] Gao YJ, Ji RR (2010) Targeting astrocyte signaling for chronic pain. Neurotherapeutics 7(4):482–493. 10.1016/j.nurt.2010.05.016 20880510 10.1016/j.nurt.2010.05.016 PMC2950097

[3] Ji RR, Donnelly CR, Nedergaard M (2019) Astrocytes in chronic pain and itch. Nat Rev Neurosci 20(11):667–685. 10.1038/s41583-019-0218-1 31537912 10.1038/s41583-019-0218-1 PMC6874831

[4] Ji RR, Nackley A, Huh Y, Terrando N, Maixner W (2018) Neuroinflammation and central sensitization in chronic and widespread pain. Anesthesiology 129(2):343–366. 10.1097/ALN.0000000000002130 29462012 10.1097/ALN.0000000000002130 PMC6051899

[5] Ji RR, Berta T, Nedergaard M (2013) Glia and pain: is chronic pain a gliopathy? Pain 154 suppl 1. 0 110.1016/j.pain.2013.06.022. S10-S28 10.1016/j.pain.2013.06.022 PMC3858488 23792284

[6] Li T, Chen X, Zhang C, Zhang Y, Yao W (2019) An update on reactive astrocytes in chronic pain. J Neuroinflammation 16(1):140. 10.1186/s12974-019-1524-2 31288837 10.1186/s12974-019-1524-2 PMC6615111

[7] Lu HJ, Gao YJ (2023) Astrocytes in chronic pain: cellular and molecular mechanisms. Neurosci Bull 39(3):425–439. 10.1007/s12264-022-00961-3 36376699 10.1007/s12264-022-00961-3 PMC10043112

[8] Chen Y, Strickland MR, Soranno A, Holtzman DM (2021) Apolipoprotein E: structural insights and links to alzheimer disease pathogenesis. Neuron 109(2):205–221. 10.1016/j.neuron.2020.10.008 33176118 10.1016/j.neuron.2020.10.008 PMC7931158

[9] Plump AS, Breslow JL (1995) Apolipoprotein E and the Apolipoprotein E-deficient mouse. Annu Rev Nutr 15:495–518. 10.1146/annurev.nu.15.070195.002431 8527231 10.1146/annurev.nu.15.070195.002431

[10] Bairamian D, Sha S, Rolhion N, Sokol H, Dorothee G, Lemere CA et al (2022) Microbiota in neuroinflammation and synaptic dysfunction: a focus on Alzheimer’s disease. Mol Neurodegener 17(1):19. 10.1186/s13024-022-00522-2 35248147 10.1186/s13024-022-00522-2 PMC8898063

[11] Fernandez-Calle R, Konings SC, Frontinan-Rubio J, Garcia-Revilla J, Camprubi-Ferrer L, Svensson M et al (2022) APOE in the Bullseye of neurodegenerative diseases: impact of the APOE genotype in Alzheimer’s disease pathology and brain diseases. Mol Neurodegener 17(1):62. 10.1186/s13024-022-00566-4 36153580 10.1186/s13024-022-00566-4 PMC9509584

[12] Parhizkar S, Holtzman DM (2022) APOE mediated neuroinflammation and neurodegeneration in Alzheimer’s disease. Semin Immunol 59:101594. 10.1016/j.smim.2022.101594 35232622 10.1016/j.smim.2022.101594 PMC9411266

[13] Islam S, Noorani A, Sun Y, Michikawa M, Zou K (2025) Multi-functional role of Apolipoprotein E in neurodegenerative diseases. Front Aging Neurosci 17:1535280. 10.3389/fnagi.2025.1535280 39944166 10.3389/fnagi.2025.1535280 PMC11813892

[14] Giau VV, Bagyinszky E, An SS, Kim SY (2015) Role of Apolipoprotein E in neurodegenerative diseases. Neuropsychiatr Dis Treat 11:1723–1737. 10.2147/NDT.S84266 26213471 10.2147/NDT.S84266 PMC4509527

[15] Tsuzuki S, Murano T, Watanabe H, Itoh Y, Miyashita Y, Shirai K (1998) [The examination of ApoE phenotypes in diabetic patients with peripheral neuropathy]. Rinsho Byori 46(8):829–833 9760837

[16] Bedlack RS, Edelman D, Gibbs JW 3rd, Kelling D, Strittmatter W, Saunders AM et al (2003) APOE genotype is a risk factor for neuropathy severity in diabetic patients. Neurology 60(6):1022–1024. 10.1212/01.wnl.0000056689.50682.94 12654974 10.1212/01.wnl.0000056689.50682.94

[17] Tansley S, Uttam S, Urena Guzman A, Yaqubi M, Pacis A, Parisien M et al (2022) Single-cell RNA sequencing reveals time- and sex-specific responses of mouse spinal cord microglia to peripheral nerve injury and links ApoE to chronic pain. Nat Commun 13(1):843. 10.1038/s41467-022-28473-8 35149686 10.1038/s41467-022-28473-8 PMC8837774

[18] Liu S, Yang S, Zhu X, Li X, Zhang X, Zhou X et al (2023) Spinal Apolipoprotein E is involved in inflammatory pain via regulating lipid metabolism and glial activation in the spinal dorsal Horn. Biol Direct 18(1):85. 10.1186/s13062-023-00444-z 38071369 10.1186/s13062-023-00444-z PMC10710718

[19] Decosterd I, Woolf CJ (2000) Spared nerve injury: an animal model of persistent peripheral neuropathic pain. Pain 87(2):149–158. 10.1016/S0304-3959(00)00276-1 10924808 10.1016/S0304-3959(00)00276-1

[20] Agalave NM, Lane BT, Mody PH, Szabo-Pardi TA, Burton MD (2020) Isolation, culture, and downstream characterization of primary microglia and astrocytes from adult rodent brain and spinal cord. J Neurosci Methods 340:108742. 10.1016/j.jneumeth.2020.108742 32315669 10.1016/j.jneumeth.2020.108742 PMC7293863

[21] Zhou K, Wei W, Yang D, Zhang H, Yang W, Zhang Y et al (2024) Dual electrical stimulation at spinal-muscular interface reconstructs spinal sensorimotor circuits after spinal cord injury. Nat Commun 15(1):619. 10.1038/s41467-024-44898-9 38242904 10.1038/s41467-024-44898-9 PMC10799086

[22] Cohen J (1992) A power primer. Psychol Bull 112(1):155–159. 10.1037//0033-2909.112.1.155 10.1037//0033-2909.112.1.155 19565683

[23] Saunders AM (2000) Apolipoprotein E and alzheimer disease: an update on genetic and functional analyses. J Neuropathol Exp Neurol 59(9):751–758. 10.1093/jnen/59.9.751 11005255 10.1093/jnen/59.9.751

[24] Smith WJ, Cedeno DL, Thomas SM, Kelley CA, Vetri F, Vallejo R (2021) Modulation of microglial activation States by spinal cord stimulation in an animal model of neuropathic pain: comparing high rate, low rate, and differential target multiplexed programming. Mol Pain 17:1744806921999013. 10.1177/1744806921999013 33626981 10.1177/1744806921999013 PMC7925954

[25] Cheng T, Xu Z, Ma X (2022) The role of astrocytes in neuropathic pain. Front Mol Neurosci 15:1007889. 10.3389/fnmol.2022.1007889 36204142 10.3389/fnmol.2022.1007889 PMC9530148

[26] Mhatre-Winters I, Eid A, Han Y, Tieu K, Richardson JR (2023) Sex and APOE genotype alter the basal and induced inflammatory States of primary astrocytes from humanized targeted replacement mice. ASN Neuro 15:17590914221144549. 10.1177/17590914221144549 36604975 10.1177/17590914221144549 PMC9982390

[27] Ren K, Torres R (2009) Role of interleukin-1beta during pain and inflammation. Brain Res Rev 60(1):57–64. 10.1016/j.brainresrev.2008.12.020 19166877 10.1016/j.brainresrev.2008.12.020 PMC3076185

[28] Leung L, Cahill CM (2010) TNF-alpha and neuropathic pain–a review. J Neuroinflammation 7:27. 10.1186/1742-2094-7-27 20398373 10.1186/1742-2094-7-27 PMC2861665

[29] Monteiro S, Roque S, Marques F, Correia-Neves M, Cerqueira JJ (2017) Brain interference: revisiting the role of IFNgamma in the central nervous system. Prog Neurobiol 156:149–163. 10.1016/j.pneurobio.2017.05.003 28528956 10.1016/j.pneurobio.2017.05.003

[30] Popko B, Corbin JG, Baerwald KD, Dupree J, Garcia AM (1997) The effects of interferon-gamma on the central nervous system. Mol Neurobiol 14(1–2):19–35. 10.1007/BF02740619 9170099 10.1007/BF02740619 PMC7091409

[31] Gajtko A, Bakk E, Hegedus K, Ducza L, Hollo K (2020) IL-1beta induced cytokine expression by spinal astrocytes can play a role in the maintenance of chronic inflammatory pain. Front Physiol 11:543331. 10.3389/fphys.2020.543331 33304271 10.3389/fphys.2020.543331 PMC7701125

[32] Van Wagoner NJ, Benveniste EN (1999) Interleukin-6 expression and regulation in astrocytes. J Neuroimmunol 100(1–2):124–139. 10.1016/s0165-5728(99)00187-3 10695723 10.1016/s0165-5728(99)00187-3

[33] Tang J, Bair M, Descalzi G (2021) Reactive astrocytes: critical players in the development of chronic pain. Front Psychiatry 12:682056. 10.3389/fpsyt.2021.682056 34122194 10.3389/fpsyt.2021.682056 PMC8192827

[34] Kawasaki Y, Zhang L, Cheng JK, Ji RR (2008) Cytokine mechanisms of central sensitization: distinct and overlapping role of interleukin-1beta, interleukin-6, and tumor necrosis factor-alpha in regulating synaptic and neuronal activity in the superficial spinal cord. J Neurosci 28(20):5189–5194. 10.1523/JNEUROSCI.3338-07.2008 18480275 10.1523/JNEUROSCI.3338-07.2008 PMC2408767

[35] Marcoli M, Cervetto C, Amato S, Fiorucci C, Maura G, Mariottini P et al (2022) Transgenic Mouse Overexpressing Spermine Oxidase in Cerebrocortical Neurons: Astrocyte Dysfunction and Susceptibility to Epileptic Seizures. Biomolecules 12(2). 10.3390/biom12020204 10.3390/biom12020204 PMC8961639 35204705

[36] Lalo U, Pankratov Y (2021) Astrocytes as perspective targets of Exercise- and caloric Restriction-Mimetics. Neurochem Res 46(10):2746–2759. 10.1007/s11064-021-03277-2 33677759 10.1007/s11064-021-03277-2 PMC8437875

[37] Kovacs Z, Skatchkov SN, Veh RW, Szabo Z, Nemeth K, Szabo PT et al (2021) Critical role of astrocytic polyamine and GABA metabolism in epileptogenesis. Front Cell Neurosci 15:787319. 10.3389/fncel.2021.787319 35069115 10.3389/fncel.2021.787319 PMC8770812

[38] Krauss M, Langnaese K, Richter K, Brunk I, Wieske M, Ahnert-Hilger G et al (2006) Spermidine synthase is prominently expressed in the striatal patch compartment and in putative interneurones of the matrix compartment. J Neurochem 97(1):174–189. 10.1111/j.1471-4159.2006.03721.x 16515550 10.1111/j.1471-4159.2006.03721.x

[39] Niechcial A, Schwarzfischer M, Wawrzyniak M, Atrott K, Laimbacher A, Morsy Y et al (2023) Spermidine ameliorates colitis via induction of Anti-Inflammatory macrophages and prevention of intestinal dysbiosis. J Crohns Colitis 17(9):1489–1503. 10.1093/ecco-jcc/jjad058 36995738 10.1093/ecco-jcc/jjad058 PMC10588784

[40] Wawrzyniak M, Groeger D, Frei R, Ferstl R, Wawrzyniak P, Krawczyk K et al (2021) Spermidine and spermine exert protective effects within the lung. Pharmacol Res Perspect 9(4):e00837. 10.1002/prp2.837 34289267 10.1002/prp2.837 PMC8294051

[41] Freitag K, Sterczyk N, Wendlinger S, Obermayer B, Schulz J, Farztdinov V et al (2022) Spermidine reduces neuroinflammation and soluble amyloid beta in an Alzheimer’s disease mouse model. J Neuroinflammation 19(1):172. 10.1186/s12974-022-02534-7 35780157 10.1186/s12974-022-02534-7 PMC9250727

[42] Zhang X, Zhao P, Kuai J, Chang C, Yuan Q (2024) [Spermidine alleviates lipopolysaccharide-induced myocardial injury in mice by suppressing apoptosis, ROS production and ferroptosis]. Nan Fang Yi Ke Da Xue Xue Bao 44(1):166–172. 10.12122/j.issn.1673-4254.2024.01.19 38293988 10.12122/j.issn.1673-4254.2024.01.19 PMC10878897

[43] Xiao L, Rao JN, Zou T, Liu L, Marasa BS, Chen J et al (2007) Polyamines regulate the stability of activating transcription factor-2 mRNA through RNA-binding protein HuR in intestinal epithelial cells. Mol Biol Cell 18(11):4579–4590. 10.1091/mbc.e07-07-0675 17804813 10.1091/mbc.E07-07-0675 PMC2043536

[44] Saha RN, Pahan K (2006) Signals for the induction of nitric oxide synthase in astrocytes. Neurochem Int 49(2):154–163. 10.1016/j.neuint.2006.04.007 16740341 10.1016/j.neuint.2006.04.007 PMC1963413

[45] Zhao ML, Liu JS, He D, Dickson DW, Lee SC (1998) Inducible nitric oxide synthase expression is selectively induced in astrocytes isolated from adult human brain. Brain Res 813(2):402–405. 10.1016/s0006-8993(98)01023-3 9838203 10.1016/s0006-8993(98)01023-3

[46] Esshili A, Manitz MP, Freund N, Juckel G (2020) Induction of inducible nitric oxide synthase expression in activated microglia and astrocytes following pre- and postnatal immune challenge in an animal model of schizophrenia. Eur Neuropsychopharmacol 35:100–110. 10.1016/j.euroneuro.2020.04.002 32439226 10.1016/j.euroneuro.2020.04.002

[47] Martens YA, Zhao N, Liu CC, Kanekiyo T, Yang AJ, Goate AM et al (2022) ApoE cascade hypothesis in the pathogenesis of Alzheimer’s disease and related dementias. Neuron 110(8):1304–1317. 10.1016/j.neuron.2022.03.004 35298921 10.1016/j.neuron.2022.03.004 PMC9035117

[48] Watanabe H, Murakami R, Tsumagari K, Morimoto S, Hashimoto T, Imaizumi K et al (2023) Astrocytic APOE4 genotype-mediated negative impacts on synaptic architecture in human pluripotent stem cell model. Stem Cell Rep 18(9):1854–1869. 10.1016/j.stemcr.2023.08.002 10.1016/j.stemcr.2023.08.002 PMC10545487 37657448

[49] Lee SI, Jeong W, Lim H, Cho S, Lee H, Jang Y et al (2021) APOE4-carrying human astrocytes oversupply cholesterol to promote neuronal lipid raft expansion and Abeta generation. Stem Cell Rep 16(9):2128–2137. 10.1016/j.stemcr.2021.07.017 10.1016/j.stemcr.2021.07.017 PMC8452535 34450034

[50] Yin Z, Rosenzweig N, Kleemann KL, Zhang X, Brandao W, Margeta MA et al (2023) APOE4 impairs the microglial response in Alzheimer’s disease by inducing TGFbeta-mediated checkpoints. Nat Immunol 24(11):1839–1853. 10.1038/s41590-023-01627-6 37749326 10.1038/s41590-023-01627-6 PMC10863749

[51] Zalocusky KA, Najm R, Taubes AL, Hao Y, Yoon SY, Koutsodendris N et al (2021) Neuronal ApoE upregulates MHC-I expression to drive selective neurodegeneration in Alzheimer’s disease. Nat Neurosci 24(6):786–798. 10.1038/s41593-021-00851-3 33958804 10.1038/s41593-021-00851-3 PMC9145692

[52] Brennan FH, Li Y, Wang C, Ma A, Guo Q, Li Y et al (2022) Microglia coordinate cellular interactions during spinal cord repair in mice. Nat Commun 13(1):4096. 10.1038/s41467-022-31797-0 35835751 10.1038/s41467-022-31797-0 PMC9283484

[53] Wang DD, Bordey A (2008) The astrocyte odyssey. Prog Neurobiol 86(4):342–367. 10.1016/j.pneurobio.2008.09.015 18948166 10.1016/j.pneurobio.2008.09.015 PMC2613184

[54] Jiang BC, Cao DL, Zhang X, Zhang ZJ, He LN, Li CH et al (2016) CXCL13 drives spinal astrocyte activation and neuropathic pain via CXCR5. J Clin Invest 126(2):745–761. 10.1172/JCI81950 26752644 10.1172/JCI81950 PMC4731172

[55] Yamasaki R, Fujii T, Wang B, Masaki K, Kido MA, Yoshida M et al (2016) Allergic inflammation leads to neuropathic pain via glial cell activation. J Neurosci 36(47):11929–11945. 10.1523/JNEUROSCI.1981-16.2016 27881779 10.1523/JNEUROSCI.1981-16.2016 PMC6604914

[56] Inoue K, Tsuda M (2018) Microglia in neuropathic pain: cellular and molecular mechanisms and therapeutic potential. Nat Rev Neurosci 19(3):138–152. 10.1038/nrn.2018.2 29416128 10.1038/nrn.2018.2

[57] Lu L, Kotowska AM, Kern S, Fang M, Rudd TR, Alexander MR et al (2024) Metabolomic and proteomic analysis of ApoE4-Carrying H4 neuroglioma cells in Alzheimer’s disease using orbisims and LC-MS/MS. Anal Chem 96(29):11760–11770. 10.1021/acs.analchem.4c01201 38989551 10.1021/acs.analchem.4c01201 PMC11270533

[58] Miranda AM, Ashok A, Chan RB, Zhou B, Xu Y, Mcintire LB et al (2022) Effects of APOE4 allelic dosage on lipidomic signatures in the entorhinal cortex of aged mice. Transl Psychiatry 12(1):129. 10.1038/s41398-022-01881-6 35351864 10.1038/s41398-022-01881-6 PMC8964762

[59] Chang R, Trushina E, Zhu K, Zaidi SSA, Lau BM, Kueider-Paisley A et al (2023) Predictive metabolic networks reveal sex- and APOE genotype-specific metabolic signatures and drivers for precision medicine in Alzheimer’s disease. Alzheimers Dement 19(2):518–531. 10.1002/alz.12675 35481667 10.1002/alz.12675 PMC10402890

[60] Madeo F, Eisenberg T, Pietrocola F, Kroemer G (2018) Spermidine in health and disease. Science 359(6374). 10.1126/science.aan2788 10.1126/science.aan2788 29371440

[61] Ni YQ, Liu YS (2021) New insights into the roles and mechanisms of spermidine in aging and Age-Related diseases. Aging Dis 12(8):1948–1963. 10.14336/AD.2021.0603 34881079 10.14336/AD.2021.0603 PMC8612618

[62] Benedikt J, Malpica-Nieves CJ, Rivera Y, Mendez-Gonzalez M, Nichols CG, Veh RW et al (2022) The Polyamine Spermine Potentiates the Propagation of Negatively Charged Molecules through the Astrocytic Syncytium. Biomolecules 12(12). 10.3390/biom12121812 10.3390/biom12121812 PMC9775384 36551240

[63] Laube G, Veh RW (1997) Astrocytes, not neurons, show most prominent staining for spermidine/spermine-like immunoreactivity in adult rat brain. Glia 19(2):171–179. 10.1002/(sici)1098-1136(199702)19:2%3C171::aid-glia8%3E3.0.co;2-3 9034833 10.1002/(sici)1098-1136(199702)19:2<171::aid-glia8>3.0.co;2-3

[64] Deng K, He H, Qiu J, Lorber B, Bryson JB, Filbin MT (2009) Increased synthesis of spermidine as a result of upregulation of arginase I promotes axonal regeneration in culture and in vivo. J Neurosci 29(30):9545–9552. 10.1523/JNEUROSCI.1175-09.2009 19641117 10.1523/JNEUROSCI.1175-09.2009 PMC6666538

[65] Yousefi-Manesh H, Shirooie S, Noori T, Sheibani M, Tavangar SM, Hemmati S et al (2023) Spermidine reduced neuropathic pain in chronic constriction injury-induced peripheral neuropathy in rats. Fundam Clin Pharmacol 37(4):779–785. 10.1111/fcp.12880 36799067 10.1111/fcp.12880

[66] Martinez-Martinez AB, Torres-Perez E, Devanney N, Del Moral R, Johnson LA, Arbones-Mainar JM (2020) Beyond the CNS: the many peripheral roles of APOE. Neurobiol Dis 138:104809. 10.1016/j.nbd.2020.104809 32087284 10.1016/j.nbd.2020.104809 PMC7195217

[67] Zhou Z, Xu MJ, Gao B (2016) Hepatocytes: a key cell type for innate immunity. Cell Mol Immunol 13(3):301–315. 10.1038/cmi.2015.97 26685902 10.1038/cmi.2015.97 PMC4856808

[68] Rebeck GW (2017) The role of APOE on lipid homeostasis and inflammation in normal brains. J Lipid Res 58(8):1493–1499. 10.1194/jlr.R075408 28258087 10.1194/jlr.R075408 PMC5538293

[69] Asante I, Louie S, Yassine HN (2022) Uncovering mechanisms of brain inflammation in Alzheimer’s disease with APOE4: application of single cell-type lipidomics. Ann N Y Acad Sci 1518(1):84–105. 10.1111/nyas.14907 36200578 10.1111/nyas.14907 PMC10092192

[70] Arnaud L, Benech P, Greetham L, Stephan D, Jimenez A, Jullien N et al (2022) APOE4 drives inflammation in human astrocytes via TAGLN3 repression and NF-kappaB activation. Cell Rep 40(7):111200. 10.1016/j.celrep.2022.111200 35977506 10.1016/j.celrep.2022.111200

[71] Choi YH, Park HY (2012) Anti-inflammatory effects of spermidine in lipopolysaccharide-stimulated BV2 microglial cells. J Biomed Sci 19(1):31. 10.1186/1423-0127-19-31 22433014 10.1186/1423-0127-19-31 PMC3320531

[72] Noro T, Namekata K, Kimura A, Guo X, Azuchi Y, Harada C et al (2015) Spermidine promotes retinal ganglion cell survival and optic nerve regeneration in adult mice following optic nerve injury. Cell Death Dis 6(4):e1720. 10.1038/cddis.2015.93 25880087 10.1038/cddis.2015.93 PMC4650557

[73] Chen Z, Lin CX, Song B, Li CC, Qiu JX, Li SX et al (2020) Spermidine activates RIP1 deubiquitination to inhibit TNF-alpha-induced NF-kappaB/p65 signaling pathway in osteoarthritis. Cell Death Dis 11(7):503. 10.1038/s41419-020-2710-y 32632306 10.1038/s41419-020-2710-y PMC7338517

