# Supplementary material for: Astrocytic spermidine insufficiency contributes to enhanced pain sensitivity associated with ApoE4
Source: J Headache Pain. 2025 May 15;26(1):116. doi: 10.1186/s10194-025-02054-8 (PMC12080267; doi:10.1186/s10194-025-02054-8)
Supplement: Supplementary file 1 — Supplementary Material 1 [file 10194_2025_2054_MOESM1_ESM.docx]

**Supplementary Figures**


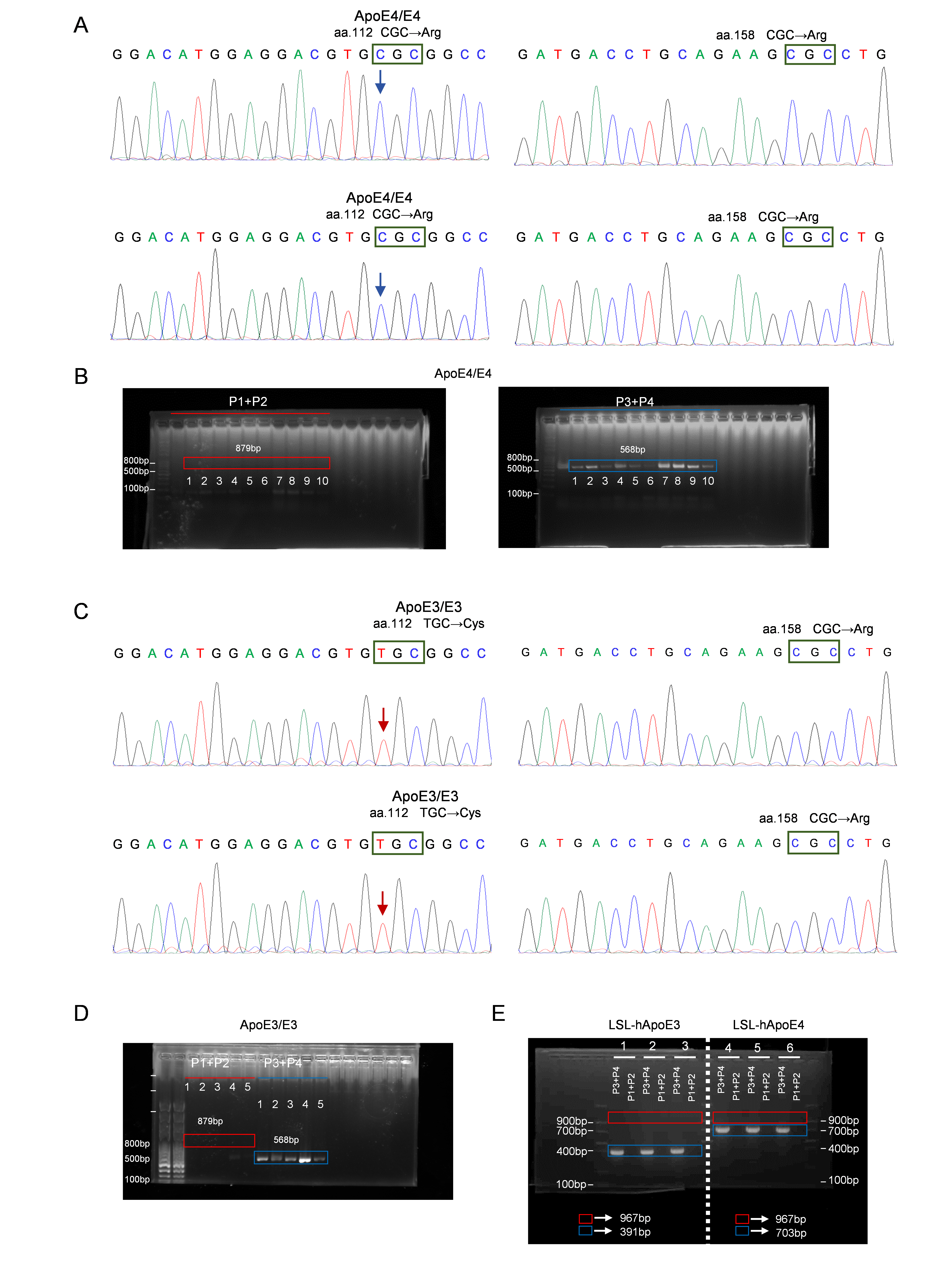


**Fig.S1 Quality control of transgenic ApoE3-TR, ApoE4-TR, LSL-hApoE3, and LSL-hApoE4 mice. (A**) Representative Sanger sequencing of ApoE4/E4 mice confirming the nucleotide substitutions at amino acid positions 112 and 158. The boxed codons indicate CGC sequences encoding arginine (Arg) at aa.112 and aa.158, which are characteristic of the ApoE4 isoform. **(B)** Genotyping results of ApoE4/E4 mice. PCR using primers P1 and P2 did not yield the 879 bp wild-type band, while PCR with primers P3 and P4 produced a 568 bp band in all samples, indicating that all mice are homozygous for the ApoE4 allele. **(C)** Representative Sanger sequencing of ApoE3/E3 mice confirming the nucleotide sequence at amino acid positions 112 and 158. The boxed codons indicate TGC (encoding cysteine, Cys) at aa.112 and CGC (encoding arginine, Arg) at aa.158, which are characteristic of the ApoE3 isoform. **(D)** Genotyping results of ApoE3/E3 mice. PCR using primers P1 and P2 did not yield the 879 bp wild-type band, while PCR with primers P3 and P4 produced a 568 bp band in all samples, indicating that all mice are homozygous for the ApoE3 allele. **(E)** Genotyping results for LSL-hApoE3 and LSL-hApoE4 mice. Left: In LSL-hApoE3 mice, PCR using primers P3 and P4 produced a 391 bp band, and no 967 bp wild-type band was detected with primers P1 and P2, confirming a homozygous genotype. Right: In LSL-hApoE4 mice, PCR using primers P3 and P4 produced a 703 bp band, and no 967 bp wild-type band was detected with primers P1 and P2, indicating a homozygous genotype.


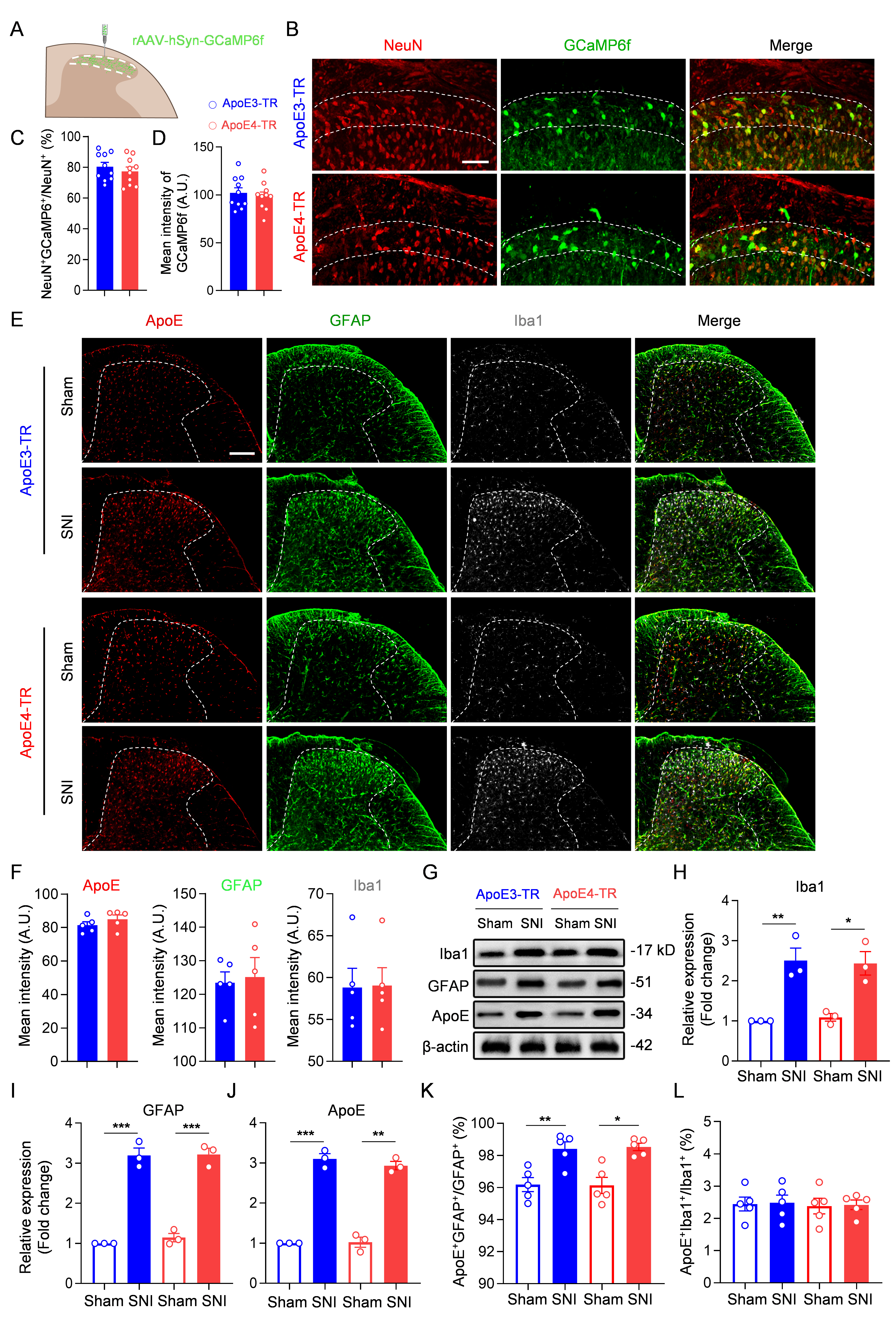


**Fig. S2** **Comparable expression of virus and glial activation between ApoE3-TR and ApoE4-TR mice. (A)** Schematic illustration of viral injection into the spinal dorsal horn. **(B)** Representative images of rAAV-hSyn-GCaMP6f expression (green) in the spinal dorsal horn of ApoE3-TR and ApoE4-TR mice, with NeuN labeling neurons (red). Scale bar, 30 μm. **(C)** Quantification of the percentage of neurons expressing GCaMP6f in the spinal dorsal horn of ApoE3-TR and ApoE4-TR mice. n = 10 mice per group. **(D)** Quantification of mean fluorescence intensity of GCaMP6f in neurons of the spinal dorsal horn at indicated groups. n = 10 mice per group. **(E)** Representative images of the spinal dorsal horn labeled by anti-ApoE (red), anti-GFAP (green), and anti-Iba1 (gray) antibodies in ApoE3-TR and ApoE4-TR mice 14 days after sham or SNI surgery. Scale bar, 100 μm. **(F)** Quantitative analysis of fluorescence intensity of ApoE, GFAP and Iba1 in the spinal dorsal horn of ApoE3-TR and ApoE4-TR mice 14 days after SNI. n = 5 mice per group. **(G)** Western blots showing the expression of Iba1, GFAP and ApoE at indicated groups. n = 3 independent biological replicates per group. **(H-J)** Quantification of relative expression of Iba1 (H), GFAP (I) and ApoE (J). **(K, L)** Quantification of the percentage of ApoE positive astrocytes (K) and ApoE positive microglia (L) in the spinal dorsal horn of ApoE3-TR and ApoE4-TR mice 14 days after sham or SNI surgery. n = 5 mice per group. All data are expressed as mean ± SEM. Statistical comparisons were conducted with one-way ANOVA followed by Tukey’s post hoc test (H-L), or unpaired Student’s t-test (C, D, F). **P* < 0.05, ***P* < 0.01, and ****P* < 0.001.


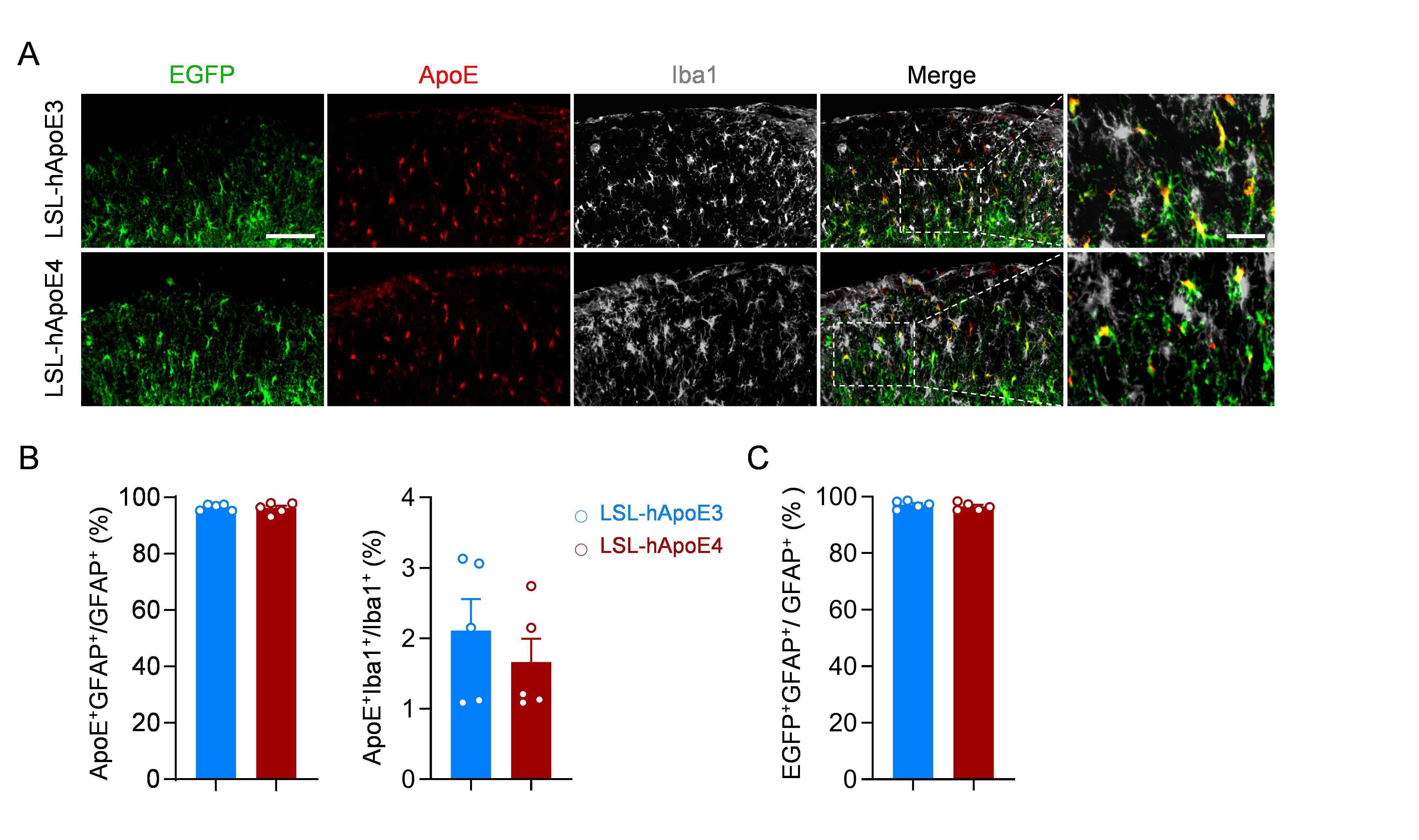


**Fig.S3 Astrocytes infected by AAV-GFAP-EGFP-Cre. (A)** Representative images of the spinal dorsal horn showing anti-ApoE (red) and anti-Iba1 (gray) staining in LSL-hApoE3 and LSL-hApoE4 mice receiving AAV-GFAP-EGFP-Cre injection. Scale bars, 50 μm (left), 10 μm (right). **(B)** Quantification of ApoE^+^GFAP^+^ cells among total GFAP^+^ cells (left) and ApoE^+^Iba1^+^ cells among total Iba1^+^ cells (right). **(C)** Quantification of EGFP^+^GFAP^+^ cells among total GFAP^+^ cells. n = 5 mice per group. All data are expressed as mean ± SEM. Statistical comparisons were conducted with unpaired Student’s t-test (B, C).


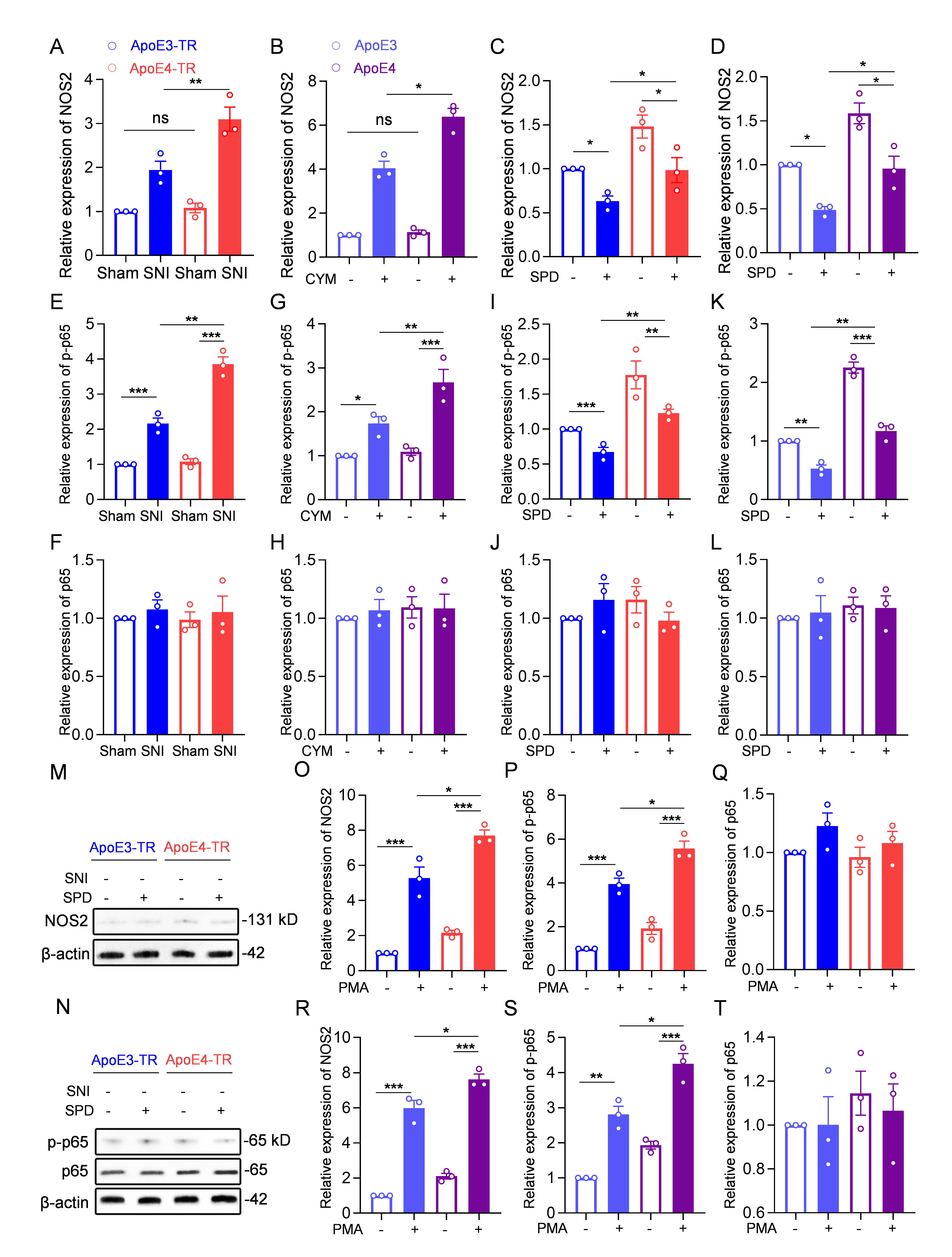


**Fig. S4 Spermidine downregulated NOS2 by inhibiting the NF-κB pathway *in vivo* and *in vitro*. (A-D**) Quantification of relative expression of NOS2 in Fig. 8A, C, E, and G. **(E-H)** Quantification of relative expression of p-p65 and p65 in Fig. 8J and K. **(I-L)** Quantification of relative expression of p-p65 and p65 in Fig. 8L and M. **(M)** Western blot analysis of NOS2 expression in the spinal dorsal horn of unoperated ApoE3-TR and ApoE4-TR mice, with or without low-dose spermidine (22.35 mM) treatment. β-actin serving as loading control. n = 3 independent biological replicates per group. **(N)** Western blot analysis of p65 and p-p65 expression in the spinal dorsal horn of unoperated ApoE3-TR and ApoE4-TR mice, with or without low-dose spermidine (22.35 mM) treatment. n = 3 independent biological replicates per group. **(O-Q)** Quantification of relative expression of NOS2, p-p65 and p65 in Fig. 8N. **(R-T)** Quantification of relative expression of NOS2, p-p65 and p65 in Fig. 8O. All data are expressed as mean ± SEM. All statistical comparisons were conducted with one-way ANOVA followed by Tukey’s post hoc test. **P* < 0.05, ***P* < 0.01 and ****P* < 0.001.
