# Supplementary material for: Astrocytic spermidine insufficiency contributes to enhanced pain sensitivity associated with ApoE4
Source: J Headache Pain. 2025 May 15;26(1):116. doi: 10.1186/s10194-025-02054-8 (PMC12080267; doi:10.1186/s10194-025-02054-8)

Figure 2B

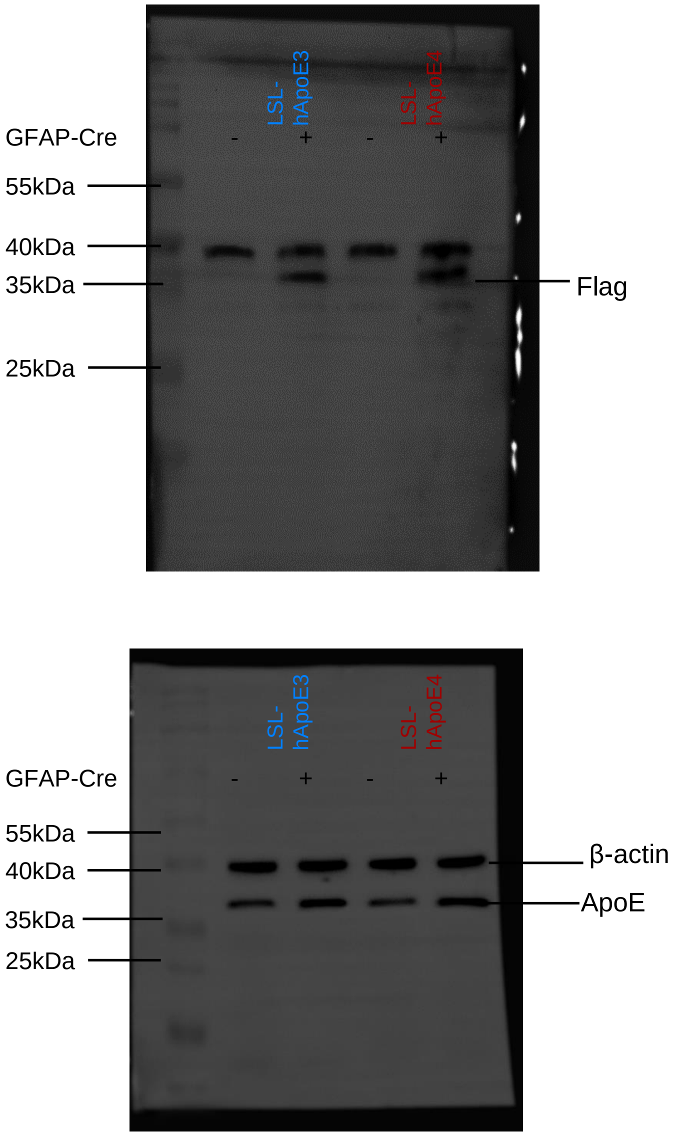
LSL-hApoE3
LSL-hApoE4
GFAP-Cre
- + - +
55kDa
40kDa
35kDa
25kDa
Flag
β-actin
ApoE

Figure 7A

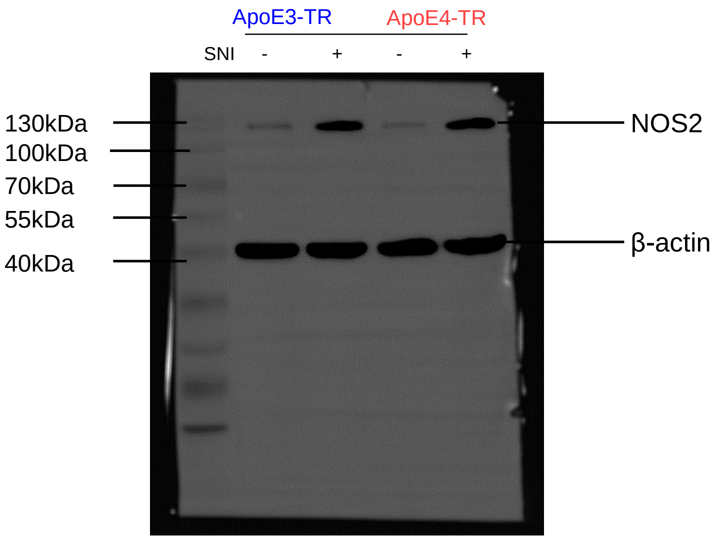

Figure 7C

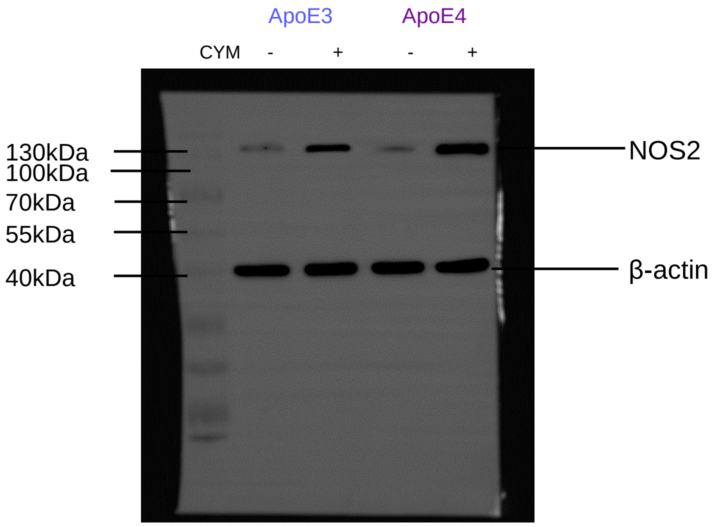

Figure 7E

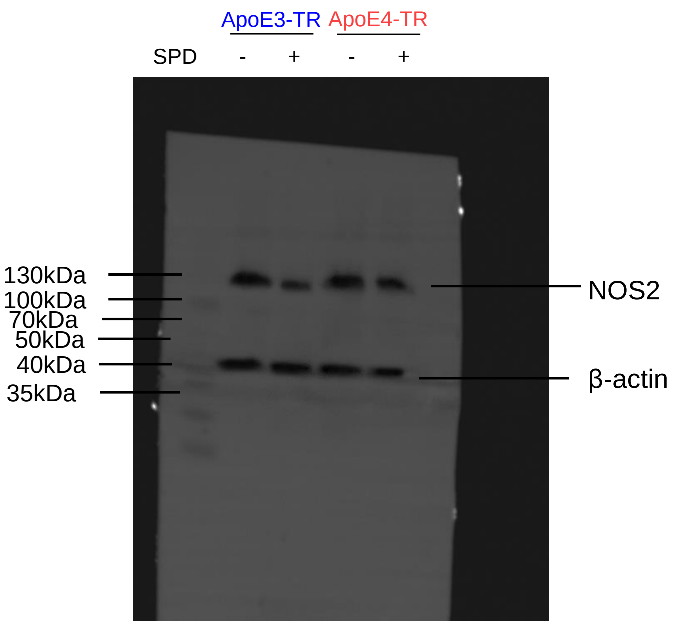

Figure 7G

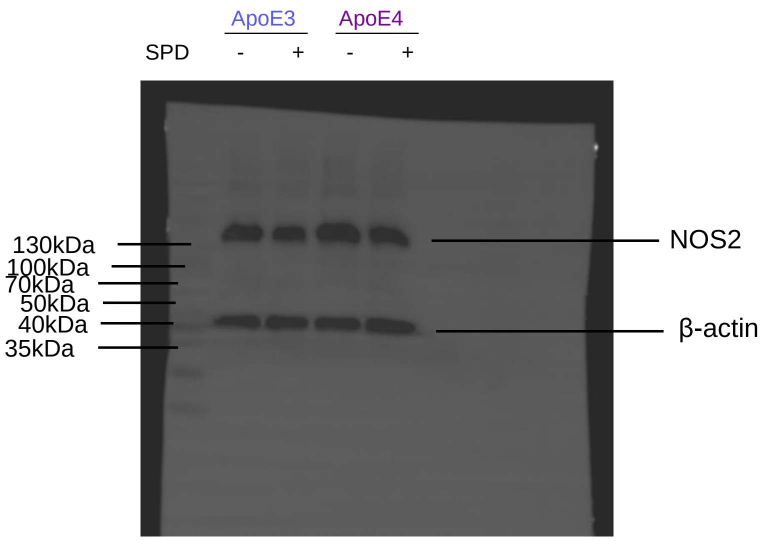

Figure 7J

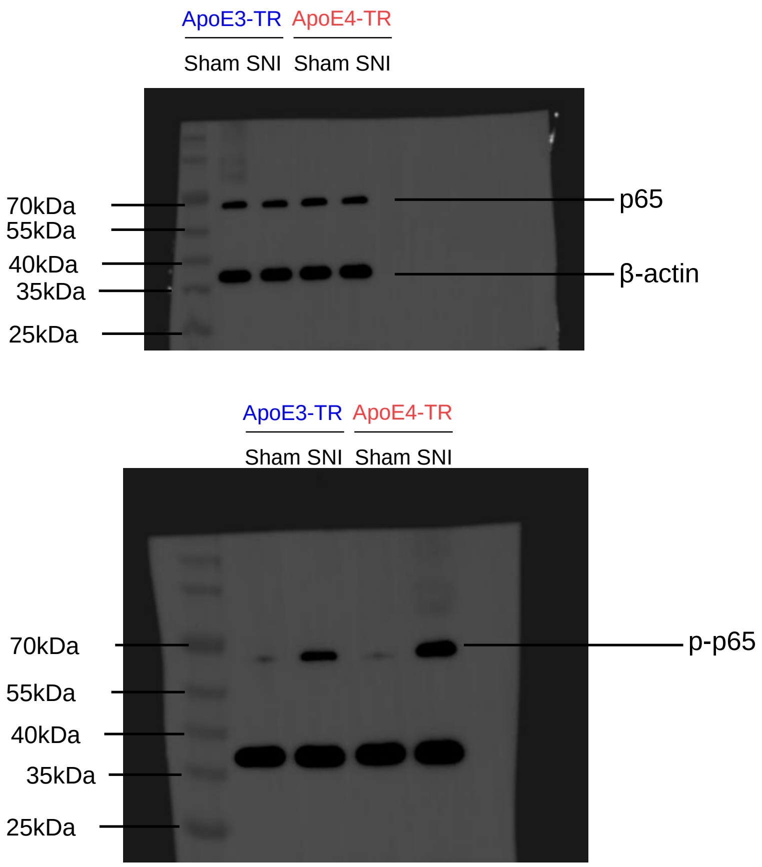

Figure 7K

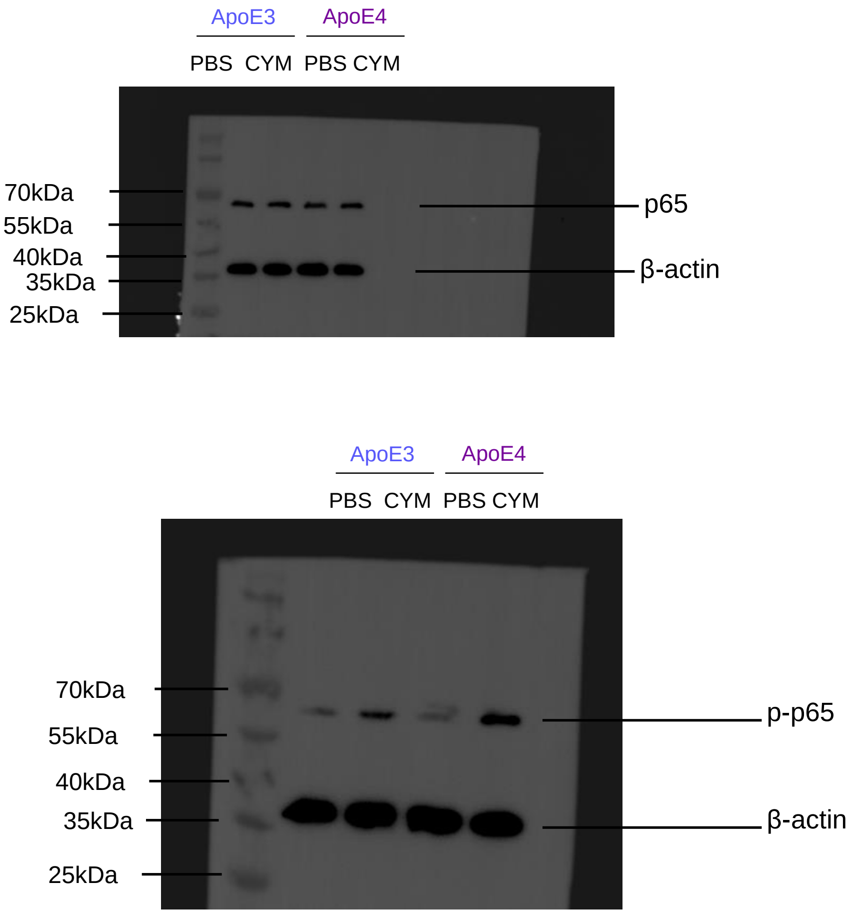

Figure 7L

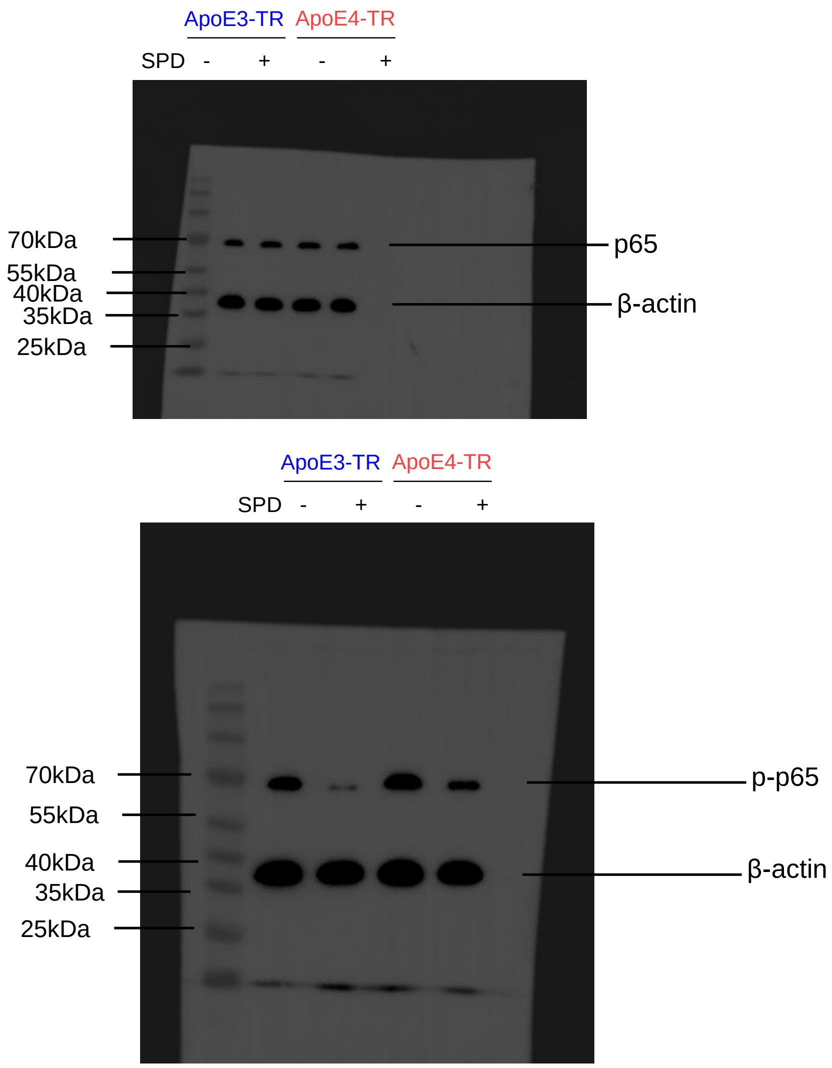

Figure 7M

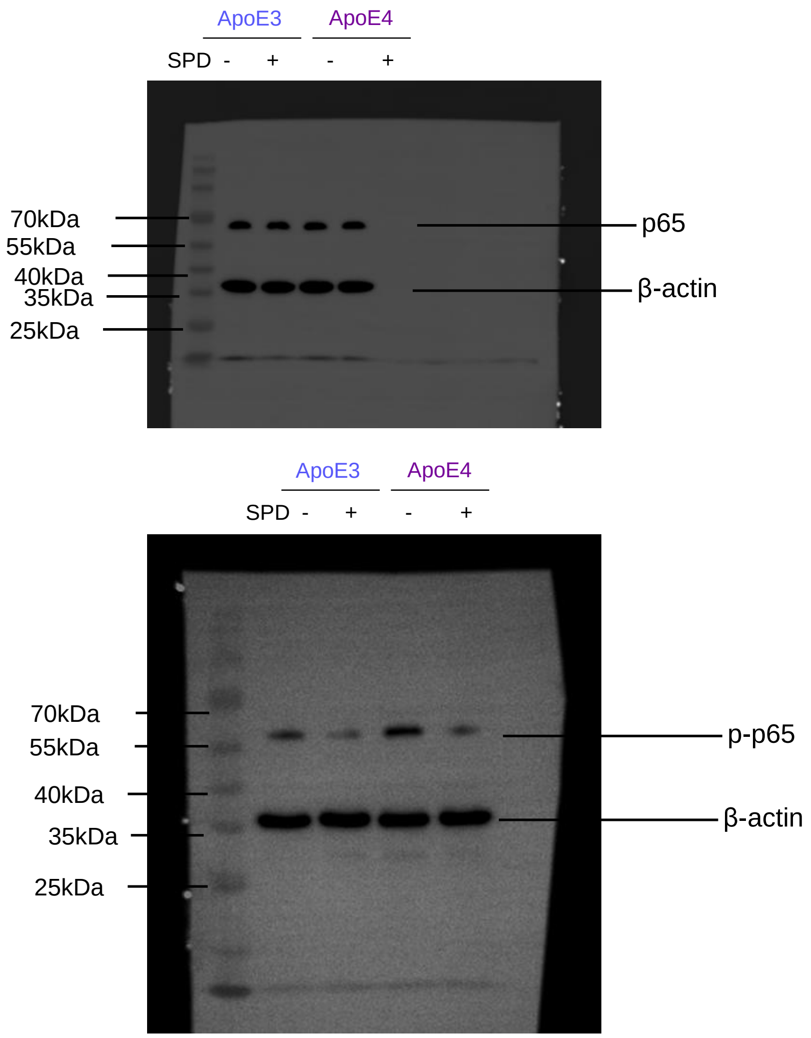

Figure 7N

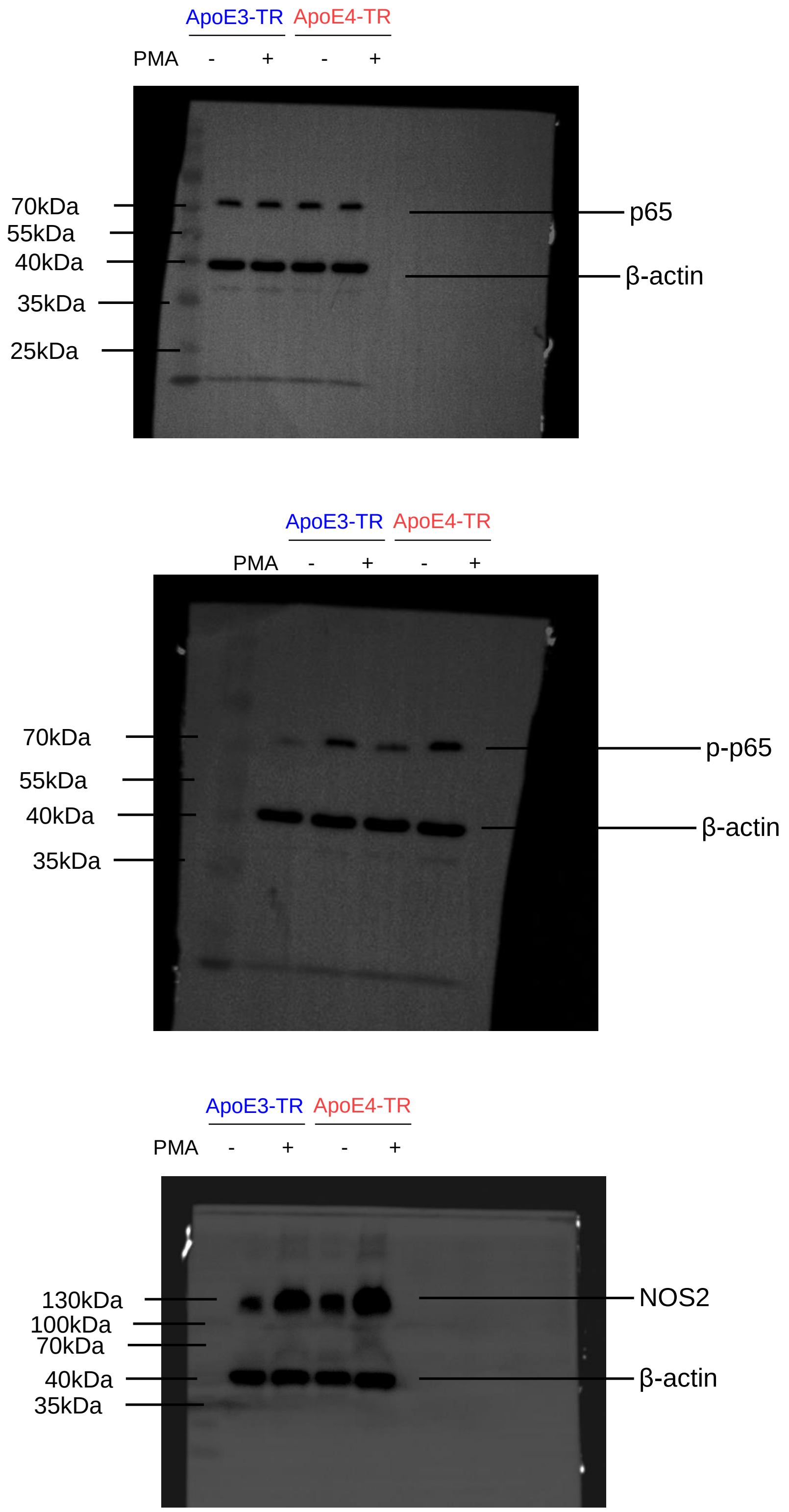

ApoE3-TR
ApoE4-TR
PMA - + - +
70kDa
55kDa
40kDa
35kDa
25kDa
p65
β-actin
ApoE3-TR
ApoE4-TR
PMA - + - +
70kDa
55kDa
40kDa
35kDa
p-p65
β-actin
ApoE3-TR
ApoE4-TR
PMA - + - +
130kDa
100kDa
70kDa
40kDa
35kDa
NOS2
β-actin

Figure 7O

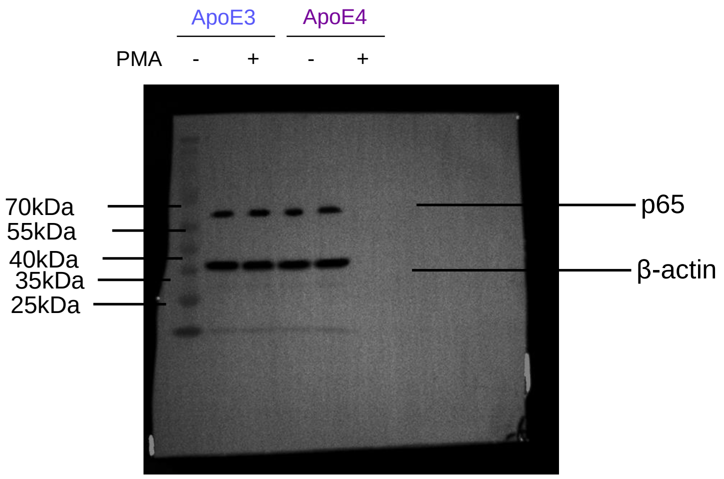
ApoE3
ApoE4
PMA - + - +
70kDa
55kDa
40kDa
35kDa
25kDa
p65
β-actin

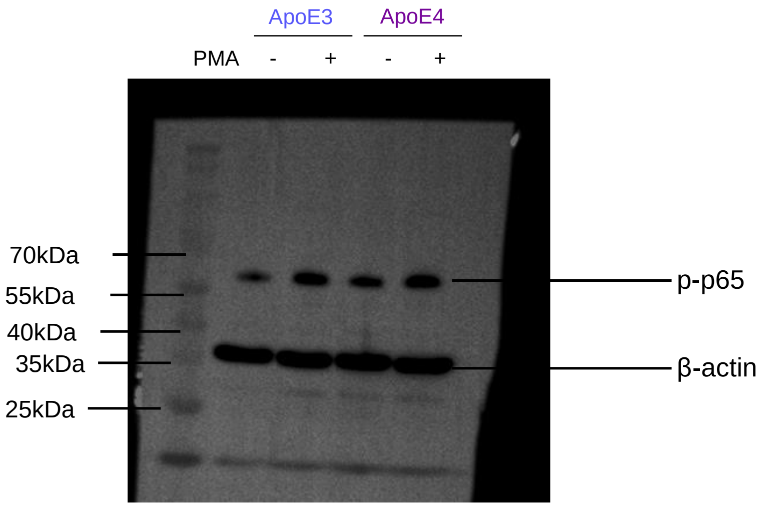
ApoE3
ApoE4
PMA - + - +
70kDa
55kDa
40kDa
35kDa
25kDa
p-p65
β-actin

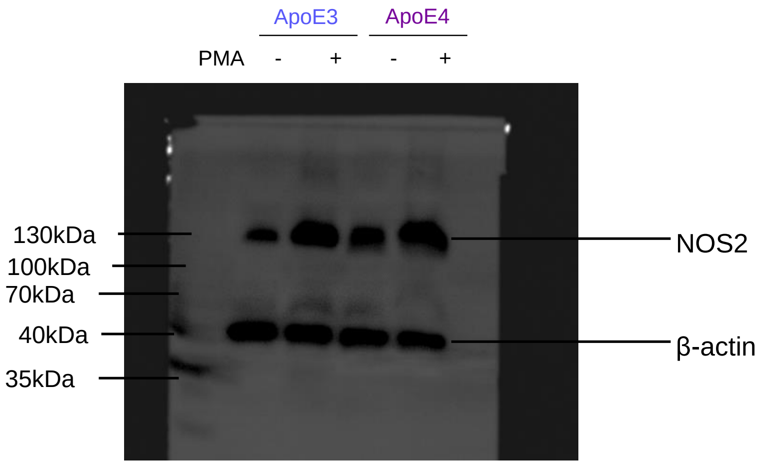
ApoE3
ApoE4
PMA - + - +
130kDa
100kDa
70kDa
40kDa
35kDa
NOS2
β-actin

Figure S2G

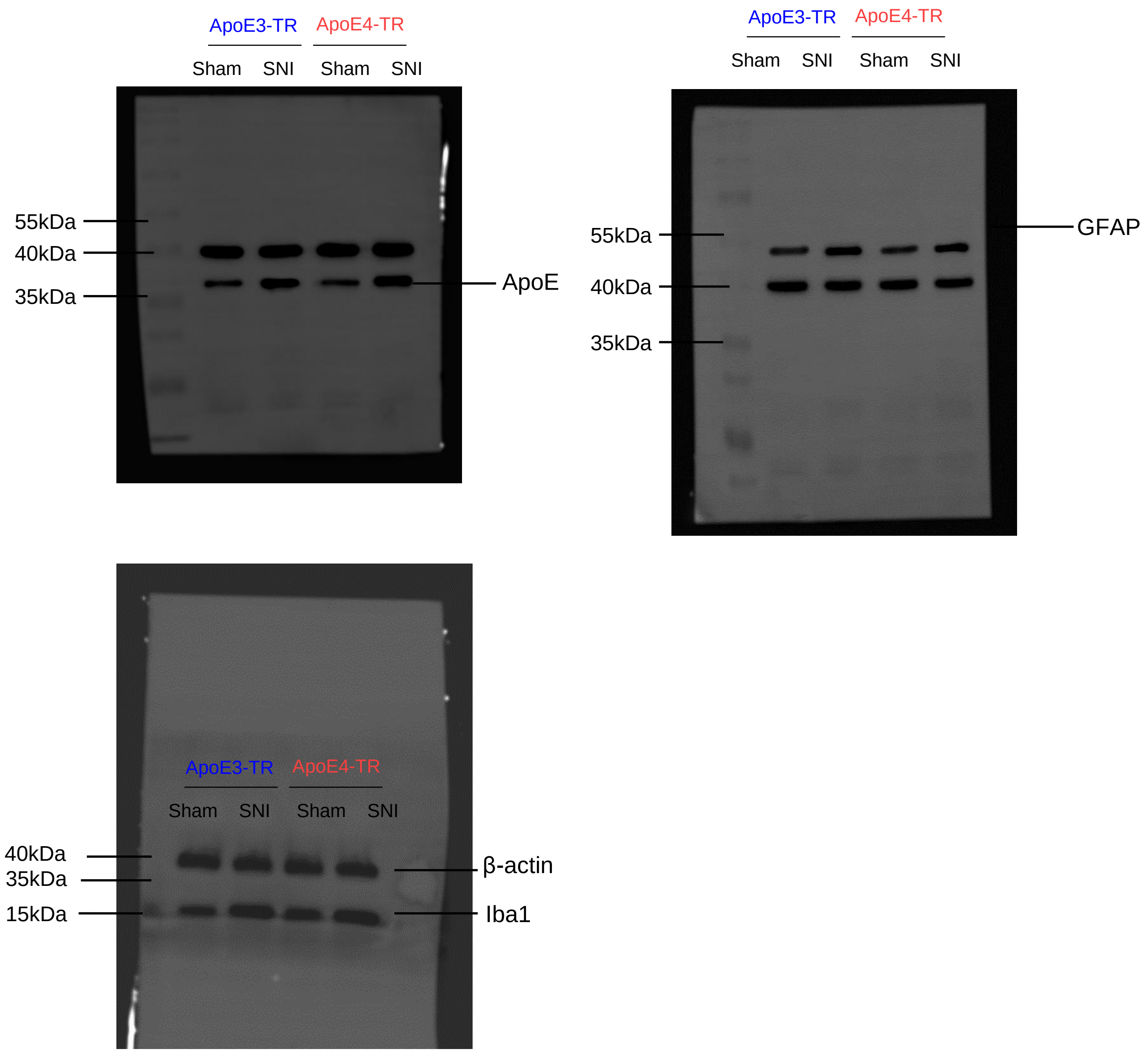

Iba1

Figure S4M

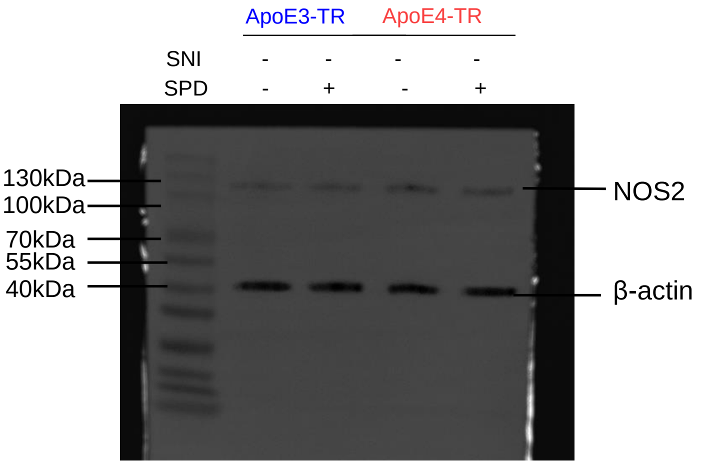

Figure S4N

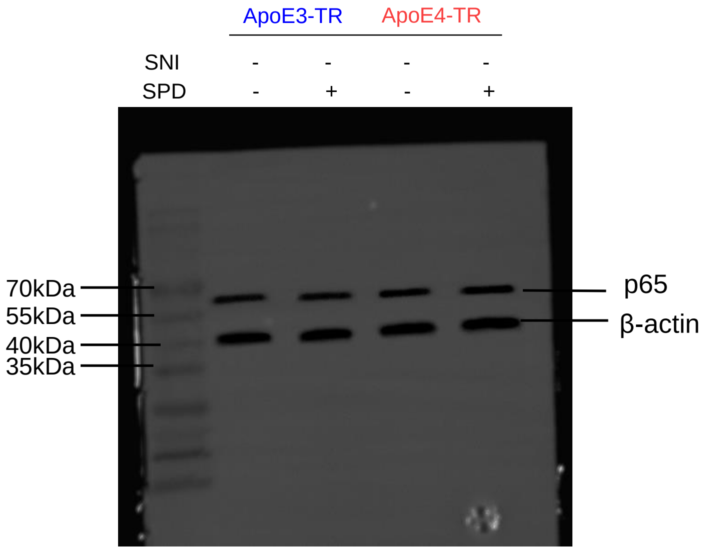

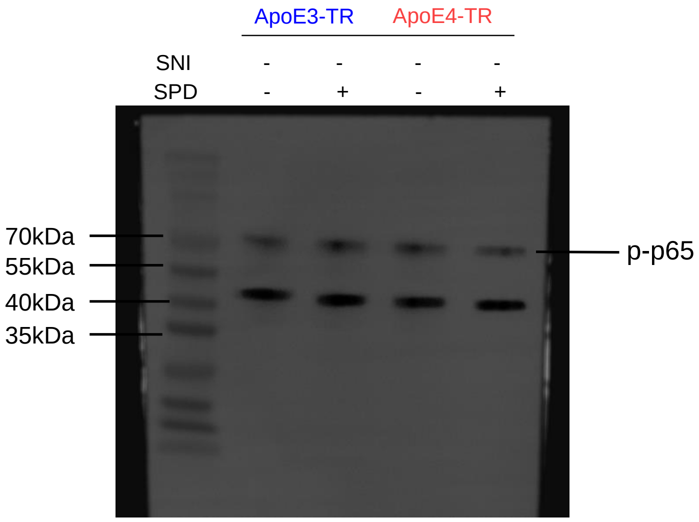

Supplement: Supplementary file 2 — Supplementary Material 2 [file 10194_2025_2054_MOESM2_ESM.pdf]
